# A general framework for comparative Bayesian meta-analysis of diagnostic studies

**DOI:** 10.1186/s12874-015-0061-7

**Published:** 2015-08-28

**Authors:** Joris Menten, Emmanuel Lesaffre

**Affiliations:** Clinical Trials Unit, Institute of Tropical Medicine, Nationalestraat 155, Antwerp, B-2000 Belgium; L-Biostat, KULeuven University of Leuven, Kapucijnenvoer 35, Leuven, B-3000 Belgium

**Keywords:** Meta-analyses, Diagnostic test accuracy, Bayesian statistics, Latent class model

## Abstract

**Background:**

Selecting the most effective diagnostic method is essential for patient management and public health interventions. This requires evidence of the relative performance of alternative tests or diagnostic algorithms. Consequently, there is a need for diagnostic test accuracy meta-analyses allowing the comparison of the accuracy of two or more competing tests. The meta-analyses are however complicated by the paucity of studies that directly compare the performance of diagnostic tests. A second complication is that the diagnostic accuracy of the tests is usually determined through the comparison of the index test results with those of a reference standard. These reference standards are presumed to be perfect, i.e. allowing the classification of diseased and non-diseased subjects without error. In practice, this assumption is however rarely valid and most reference standards show false positive or false negative results. When an imperfect reference standard is used, the estimated accuracy of the tests of interest may be biased, as well as the comparisons between these tests.

**Methods:**

We propose a model that allows for the comparison of the accuracy of two diagnostic tests using direct (head-to-head) comparisons as well as indirect comparisons through a third test. In addition, the model allows and corrects for imperfect reference tests. The model is inspired by mixed-treatment comparison meta-analyses that have been developed for the meta-analysis of randomized controlled trials. As the model is estimated using Bayesian methods, it can incorporate prior knowledge on the diagnostic accuracy of the reference tests used.

**Results:**

We show the bias that can result from using inappropriate methods in the meta-analysis of diagnostic tests and how our method provides more correct estimates of the difference in diagnostic accuracy between two tests. As an illustration, we apply this model to a dataset on visceral leishmaniasis diagnostic tests, comparing the accuracy of the RK39 dipstick with that of the direct agglutination test.

**Conclusions:**

Our proposed meta-analytic model can improve the comparison of the diagnostic accuracy of competing tests in a systematic review. This is however only true if the studies and especially information on the reference tests used are sufficiently detailed. More specifically, the type and exact procedures used as reference tests are needed, including any cut-offs used and the number of subjects excluded from full reference test assessment. If this information is lacking, it may be better to limit the meta-analysis to direct comparisons.

**Electronic supplementary material:**

The online version of this article (doi:10.1186/s12874-015-0061-7) contains supplementary material, which is available to authorized users.

## Background

There is a growing interest in diagnostic test accuracy (DTA) reviews to select the best diagnostic test procedure [1] for a given setting. Most meta-analyses of diagnostic tests, however, estimate the diagnostic accuracy of a single test [2, 3]. Selection of the best test is usually done by undertaking separate meta-analyses for each test and then comparing the results [3]. Even when formally comparing diagnostic tests in a single systematic review, the analysis may ignore study effects. Such an approach can lead to biased comparisons due to confounding by study effects, as shown in a recent review [3]. Takwoingi and colleagues showed that results from comparative studies, where two tests were directly compared and which provide the most robust comparisons, differed from those of non-comparative studies. However, only 31 % of available studies were comparative. This indicates that there is a need for meta-analytical methods via direct and indirect comparisons. Data from direct comparisons may be inconclusive while a combined analysis of direct and indirect comparisons may be conclusive and can result in more accurate estimates [4, 5].

A particular aspect of comparisons between diagnostic tests is that the diagnostic performance of the index test is nearly always determined by comparison with a second test, the reference standard. Such a reference standard is presumed to 100 % correctly classify subjects as diseased or not. However, for many diseases it is impossible to determine the true disease status with certainty [6] and reference standards are imperfect. It is well known that the use of imperfect reference standards may bias estimates of the accuracy of the index test [7]. This consideration leads to a second requirement for comparative meta-analyses of diagnostic studies: the meta-analytic methods should adjust for the use of imperfect reference standards.

The aim of this manuscript is to develop a model that can be used for the comparative meta-analysis of two diagnostic tests that conforms to the two requirements sketched above. First, we assess possible biases in the estimation of the relative accuracy of two index tests due to the use of imperfect reference tests. We describe the different parameters that can be used to estimate the relative accuracy of two tests and assess the bias resulting from the use of imperfect reference standards. This allows us to select the most appropriate summary measure to use in the comparative meta-analysis of two diagnostic tests. Subsequently, we describe and develop models that can be used in the meta-analysis of diagnostic studies to compare the relative accuracy of two tests. We start with models that presume a perfect reference test is used in each primary study and extend these models allowing for imperfect reference tests. We estimate these models using Bayesian methods, specifically using Markov-Chain Monte-Carlo (MCMC) methods through Gibbs sampling [8]. For each model we provide the model specification and offer suggestions for appropriate informative or vague priors. In addition, we assess in a simulation study the value of these newly developed models but also the bias induced by the use of incorrect methods. Finally, we apply the methods to a real data example in the field of leishmaniasis.

## Methods

Our aim is to estimate and test the difference in diagnostic accuracy of two or more index tests in a meta-analysis, combining data across all available studies. The studies included in a DTA review typically test each subject with one or more index tests and with one reference test. This reference test may differ between studies. To set the scene, data from a hypothetical meta-analysis are presented in Table 1. In this example, there are three index tests (*T*_1_,*T*_2_,*T*_3_) and two possible reference tests (*T*_4_,*T*_5_). For example, in Study 1 index tests *T*_1_ and *T*_2_ are performed on all subjects as well as reference test *T*_4_. There are 30 subjects with positive results on all three tests, one subject shows positive results on *T*_1_ and *T*_2_ and a negative result on *T*_4_, etc. Studies 1 and 2 allow direct estimation of the relative accuracy of *T*_1_ and *T*_2_. Studies 3, 4 and 5 allow the estimation of the accuracy of *T*_1_ (studies 3 and 4) or *T*_2_ (study 5), but allow no direct comparison of *T*_1_ and *T*_2_. For these studies, the relative accuracy of *T*_1_ and *T*_2_ can only be estimated by estimating the diagnostic accuracy of each test separately and then comparing these estimates. This is complicated by the fact that the reference test is not the same for each study. Studies 6 and 7 do not allow direct comparison of the accuracy *T*_1_ and *T*_2_, but offer the possibility of an indirect comparison through the third index test *T*_3_. The information from this third test may help to eliminate differences among the studies.

**Table 1:** Tabulation of an hypothetical diagnostic test accuracy meta-analysis. Columns *T*_1_,*T*_2_,*T*_3_ indicate results for the 3 possible index tests. Columns *T*_4_,*T*_5_ indicate results for the 2 possible reference tests. + indicates a positive test result, - a negative test result. NA indicates that the test was not performed in that particular study. The observed frequency column report the number of subjects with a specific test result pattern in each study

Study	Index tests			Reference		Observed
Nr.	*T*_1_	*T*_2_	*T*_3_	*T*_4_	*T*_5_	Frequency
1	+	+	NA	+	NA	30
1	+	+	NA	-	NA	1
1	+	-	NA	+	NA	3
1	+	-	NA	-	NA	6
1	-	+	NA	+	NA	0
1	-	+	NA	-	NA	3
1	-	-	NA	+	NA	8
1	-	-	NA	-	NA	160
2	+	+	+	NA	+	49
…	…	…	…	…	…	…
2	-	-	-	NA	-	99
3	+	NA	NA	+	NA	115
…	…	…	…	…	…	…
3	-	NA	NA	-	NA	244
4	+	NA	NA	NA	+	11
…	…	…	…	…	…	…
4	-	NA	NA	NA	-	19
5	NA	+	NA	+	NA	66
…	…	…	…	…	…	…
5	NA	-	NA	-	NA	29
6	+	NA	+	NA	+	27
…	…	…	…	…	…	…
6	-	NA	-	NA	-	56
7	NA	+	+	NA	+	77
…	…	…	…	…	…	…
7	NA	-	-	NA	-	13
8	+	+	+	NA	NA	143
…	…	…	…	…	…	…
8	-	-	-	NA	NA	85

As a first step in a comparative DTA meta-analysis, we have to select an appropriate statistic to compare the two tests. The best statistic would be one which is readily interpretable by users of the meta-analysis and which is least prone to bias. We describe the possible choices below together with the results from a small simulation study. Subsequently, we need to develop a model which allows the incorporation of all available data while ensuring that results are valid and are not biased by differences in study characteristic, such as the selection of the reference standard used. Some possible models are described below. We assessed the value of these models in a simulation study and in a practical application.

### Measures of relative value of diagnostic tests

Diagnostic accuracy is characterised by sensitivity *S* and specificity *C*. These two quantities are related and comparisons between tests need to take both *S* and *C* into account. Comparisons between two tests can be summarized using the difference or relative risk in *S* and *C* for the two tests. An alternative parameterization uses the diagnostic odds ratio *DOR*=(*S*×*C*)/[(1−*S*)×(1−*C*)] which summarizes the accuracy of a test in a single number [9]. This parameter could be used as a summary in a meta-analysis, for example by calculating the relative DOR of two tests [3]. However, the use of imperfect reference standards can bias all above measures of relative accuracy of two index tests. We assessed the direction and magnitude of bias on these measures in a small simulation study. A description of the simulation study setup is given in Additional file 1.

### Models for the comparative meta-analysis of diagnostic tests

In this section we develop models to compare *J* tests, by combining data across *I* studies in a comparative meta-analysis. All these models are hierarchical in nature. At the first level of the hierarchy, the models describe the observed data of the individual studies. The observed test outcomes depend on the disease prevalence and the accuracy of the tests in each study, and possible covariation among the test results. We describe the accuracy of the tests in terms of the study-specific sensitivity *S*_*ij*_ and specificity *C*_*ij*_ of test *j* in study *i*. At the second level, we specify a model for these study-specific sensitivity-specificity pair {*S*_*ij*_,*C*_*ij*_}. Five possible models are described; they are listed in Table 2.

**Table 2:** Description of the different models. Example code for the models is given in Additional file 2. *S*_*ij*_ and *C*_*ij*_ represent the sensitivity and specificity of test *j* in study *i*

Model	Reference standard	Model estimation
1	Assumed to be perfect	Independent estimation of *S*_*ij*_ and *C*_*ij*_
2	Assumed to be perfect	Direct comparisons only
3	Assumed to be perfect	Direct and indirect comparisons
4	Allowing for imperfect reference standards	Hierarchical latent class model
5	Allowing for imperfect reference standards	Network-based latent class model

#### Meta-analytic models when a perfect reference standard is available

If a perfect reference test is available, the number of diseased *N*_*Di*_ and non-diseased *N*_*NDi*_ subjects in study *i* is known, as are the numbers of true positives *N*_*TPij*_ and true negatives *N*_*TNij*_ for each test *j*. In the standard bivariate model for the meta-analysis of a diagnostic test [9], the observed numbers of true positives and true negatives for each index test are assumed to be drawn from two independent binomial distributions *N*_*TPij*_∼*Bin*(*N*_*Di*_,*S*_*ij*_) and *N*_*TNij*_∼*Bin*(*N*_*NDi*_,*C*_*ij*_). The transformed values *g*(*S*_*ij*_) = *θ*_*Sij*_ and *g*(*C*_*ij*_) = *θ*_*Cij*_ are modeled at the next level, where *g*(.) is a link function to allow the use of the normal distribution. Common choices for *g*(.) are the logit, complementary log-log or probit functions. Several models are possible to incorporate comparisons of the diagnostic accuracy of different tests in this framework. We discuss three models below. These models can be further expanded to allow for covariates, other dependence structures or alternative parameterizations.

##### *Model 1: Standard bivariate model for the meta-analysis of diagnostic tests*

A basic approach is to estimate the average diagnostic accuracy of each test separately and subsequently compare the estimates of the average *S*_*j*_ and *C*_*j*_ across the different studies. In this approach, the standard bivariate model for the meta-analysis of diagnostic tests [2] can be used for each test separately. All *g*(*S*_*ij*_)=*θ*_*Sij*_ and *g*(*C*_*ij*_)=*θ*_*Cij*_ pairs are assumed to follow independent bivariate normal distributions:

(1) $$\begin{array}{*{20}l} \left(\begin{aligned} \theta_{Sij} \\ \theta_{Cij} \end{aligned} \right) \sim N \left(\left[ \begin{aligned} \mu_{S_{j}} \\ \mu_{C_{j}} \end{aligned} \right], \Sigma_{j} \right), \\ \text{with} \ \Sigma_{j} = \left(\begin{aligned} & \sigma^{2}_{S_{j}} & \sigma_{S_{j}C_{j}} \\ & \sigma_{S_{j}C_{j}} & \sigma^{2}_{C_{j}} \end{aligned} \right),  \end{array} $$

where $\rho _{S_{j}C_{j}}=\sigma _{S_{j}C_{j}}/(\sigma _{S_{j}}\times \sigma _{C_{j}})$ is the correlation between $\theta _{S_{\textit {ij}}}$ and $\theta _{C_{\textit {ij}}}$. Estimates of the relative accuracy of the tests are obtained from the estimated $g^{-1}(\mu _{S_{j}})$ and $g^{-1}(\mu _{C_{j}})$. For example, the average difference in *S* between *T*_1_ and *T*_2_ is estimated as $\hat {S}_{D21} = g^{-1}(\hat {\mu }_{S_{2}}) - g^{-1}(\hat {\mu }_{S_{1}})$. The advantage of the standard bivariate model is that it is relatively easy to fit using both Bayesian or frequentist techniques, with SAS [9] and WinBUGS [10, 11] example code available. However, as this model is not based on the comparisons between the index tests, but on the pooling of results for each test across all available studies, the results may be biased by study characteristics. This is equivalent to pooling findings from the active treatment arms of RCTs and comparing these estimates, an approach which is considered not to be appropriate for the meta-analysis of RCTs [12].

##### *Model 2: Meta-Analysis Based on Direct Comparisons*

To take study effects into account, the overall probability of testing positive in diseased subjects *μ*_*Si*_ or in non-diseased subjects *μ*_*Ci*_ for each study *i* could be modeled and *S*_*ij*_ and *C*_*ij*_ of the individual tests described as contrasts from this overall probability.

If we limit the data to studies which compare the two tests directly, we can write the study specific, transformed sensitivities *g*(*S*_*ij*_) = *θ*_*Sij*_ and specificities *g*(*C*_*ij*_) = *θ*_*Cij*_ as follows:

(2) $$ \begin{aligned} \theta_{Si1} = \mu_{Si} + \delta_{Si}/2, \\ \theta_{Si2} = \mu_{Si} - \delta_{Si}/2, \\ \theta_{Ci1} = \mu_{Ci} - \delta_{Ci}/2, \\ \theta_{Ci2} = \mu_{Ci} + \delta_{Ci}/2. \end{aligned}   $$

In case *g* is the logit function, *δ*_*Si*_= log(*S*_*OR*12_) and *δ*_*Ci*_= log(*C*_*OR*12_), i.e. the log of the ORs of testing positive in diseased subjects for *T*_1_ compared to *T*_2_ and the log of the ORs of testing negative in non-diseased subjects in study *i*, respectively. To obtain average estimates of the difference in diagnostic accuracy between the two tests, *δ*_*Si*_ and *δ*_*Ci*_ are modeled using a bivariate normal distribution:

(3) $$\begin{array}{*{20}l} \left(\begin{aligned} \delta_{Si} \\ \delta_{Ci} \end{aligned} \right) \sim N \left(\left[ \begin{aligned} \nu_{\delta_{S}} \\ \nu_{\delta_{C}} \end{aligned} \right], \Sigma \right) \\ \text{with} \ \Sigma = \left(\begin{aligned} & \sigma^{2}_{\delta_{S}} & \sigma_{\delta_{S}\delta_{C}} \\ & \sigma_{\delta_{S}\delta_{C}} & \sigma^{2}_{\delta_{C}} \end{aligned} \right).  \end{array} $$

The $\nu _{\delta _{S}}$ and $\nu _{\delta _{C}}$ are the average log OR of the *S* and *C* between tests *T*_1_ and *T*_2_, respectively. The *μ*_*Si*_ and *μ*_*Ci*_ account for the dependence of test results obtained from the same study and can be estimated as fixed effects of in their turn modeled using bivariate normal distributions. This model is equivalent to the Smith −*Spiegelhalter*−Thomas model for two-treatment comparisons of RCTs [13, 14]. A similar model, but assuming a fixed, rather than random, relative accuracy between the different index tests is described in the Cochrane Handbook for Systematic Reviews of DTA studies [9].

##### *Model 3: Meta-Analysis Based on Direct and Indirect Comparisons*

As shown in Lu et al. [14] in the case of meta-analysis of RCTs, the Smith −*Spiegelhalter*− Thomas model can be expanded to a mixed treatment-comparison meta-analysis of more than two treatments. Similarly, we can expand Model 2 to *J* diagnostic tests. By taking diagnostic test *T*_*J*_ as baseline, we can rewrite eqs. 2 and 3 as:

$$\begin{aligned} \theta_{Si1} = \mu_{Si} + (\,J-1) \times \delta_{Si1}/J - \delta_{Si2}/J - \ldots - \delta_{Si(\,J-1)}/J, \\ \theta_{Si2} = \mu_{Si} - \delta_{Si1}/J + (\,J-1) \times \delta_{Si2}/J - \ldots - \delta_{Si(\,J-1)}/J, \\ \vdots \\ \theta_{SiJ} = \mu_{Si} - \delta_{Si1}/J - \delta_{Si2}/J - \ldots - \delta_{Si(\,J-1)}/J, \end{aligned} $$

$$\begin{aligned} \theta_{Ci1} = \mu_{Ci} + (\,J-1) \times \delta_{Ci1}/J - \delta_{Ci2}/J - \ldots - \delta_{Ci(\,J-1)}/J, \\ \theta_{Ci2} = \mu_{Ci} - \delta_{Ci1}/J + (\,J-1) \times \delta_{Ci2}/J - \ldots - \delta_{Ci(\,J-1)}/J, \\ \vdots \\ \theta_{CiJ} = \mu_{Ci} - \delta_{Ci1}/J - \delta_{Ci2}/J - \ldots - \delta_{Ci(\,J-1)}/J, \end{aligned} $$

with

(4) $$ \left(\delta_{Si1}, \delta_{Si2}, \ldots, \delta_{Si(\!\,J-1)}, \delta_{Ci1}, \delta_{Ci2}, \ldots, \delta_{Ci(\,J-1)} \right) \sim \!N(\boldsymbol{\nu_{\delta}},\Sigma).   $$

and $\phantom {\dot {i}\!}\boldsymbol {\nu }_{\boldsymbol {\delta }}=\left (\nu _{\delta _{S1}},\ldots,\nu _{\delta _{S(\,J-1)}}, \nu _{\delta _{C1}},\ldots,\nu _{\delta _{C(\,J-1)}}\right)$ represents the average log ORs for *S* and *C* of the *J*−1 tests compared to the baseline test *T*_*J*_. The differences in *S* and *C* between *T*_1_ and *T*_2_ on the logit scale are estimated by $\nu _{\delta _{S1}} - \nu _{\delta _{S2}}$ and $\nu _{\delta _{C1}} - \nu _{\delta _{C2}}$, respectively. This method allows indirect comparisons of *T*_1_ and *T*_2_ through comparison with a third test, similar to mixed treatment comparisons meta-analysis of RCTs. One complication of this model, is the specification and estimation of the variance-covariance matrix *Σ*. Specifying a structured variance-covariance matrix is in general complex and difficult to handle in MCMC estimation since each sampled variance-covariance matrix should be positive-definite [15]. In addition, model identification of the model with a general variance-covariance matrix will be difficult, especially when number of tests of interest is large. As an initial exploration of this model we can use a simplified variance-covariance structure, for example a diagonal or block diagonal matrix, and subsequently assess the effects of relaxing the simplifying assumptions. We describe some possible simplified variance-covariance structures in Additional file 2: Section 2.6.

#### Meta-analytic models when no perfect reference standard is available

##### *Introduction*

The models described above presume that the disease status of all subjects in all studies is known, and consequently that the *N*_*Di*_,*N*_*NDi*_,*N*_*TPij*_ and *N*_*TNij*_ for each study *i* and test *j* is available. However, if only imperfect reference standards are available, the reported estimates of these quantities may be biased. The models described above can be expanded through latent class analysis (LCA) [16] to allow for the use of imperfect reference standards. In LCA, the true disease status of the participants of the basic studies is an unobserved, or latent, variable with two mutually exclusive categories, “diseased” and “non-diseased”. This unobserved variable determines the probability to test positive or negative to a number of diagnostic tests which may include one or more imperfect reference tests. LCA models have been described for a variety of situations ranging from when a single imperfect test is observed in each study to more complex designs involving multiple tests. When multiple tests are involved, they may be treated as independent conditional on the disease status or the conditional dependence between them may be modeled using a variety of approaches [17–20]. An important underlying assumption of the latent class model is that the tests included in the model all correspond to the same underlying disease state [21]. Especially in a meta-analysis, where each study may use a different set of tests, this assumption is critical. If this assumption is not met, the underlying latent variable may differ among studies.

##### *Description of the conditional independence latent class model*

In this section, we describe the basic latent class model at the level of the individual study *i* in the meta-analysis. To simplify notation, we temporarily suppress the *i*-subscript for the study level. For latent class analysis, the basic data is not the number of true positives and true negatives for each test *j*, but rather the number of subjects that show a certain pattern of outcomes across the *J* tests performed in a study. The number of subjects with pattern **y**=(*y*_1_,*y*_2_,…,*y*_*j*_) can be denoted as *N*_**y**_ and is assumed to follow a multinomial distribution *N*_**y**_∼Mult[*N*,*P*(**y**)], with *y*_*j*_ the observed binary outcome (0 = negative, 1 = positive) for test *T*_*j*_, *N* the total sample size and *P*(**y**) the probability that **y** occurs.

Denoting the unobserved disease status as *D* (not diseased *D*=0, diseased *D*=1) and under the conditional independence assumption $P(\mathbf {y}|D=k) = \prod _{j=1}^{J} P\left (y_{j}|D=k\right)$, the class probabilities *P*(**y**) can be described in terms of the *S*_*j*_ and *C*_*j*_ of the *J* tests. That is:

(5) $$\begin{array}{@{}rcl@{}} P\left(\mathbf{y}\right)= \sum_{k=0}^{1} P\left(D=k\right) P\left(\mathbf{y}|D=k\right) =  \\ {}\pi \prod_{j=1}^{J} S^{y_{j}}_{j} \left(1-S_{j}\right)^{\left(1-y_{j}\right)}\,+\, \left(1-\pi\right) \prod_{j=1}^{J} C^{\left(1-y_{j}\right)}_{j} \left(1\,-\,C_{j}\right)^{y_{j}}, \end{array} $$

with *π* the disease prevalence.

Thus LCA provides estimates for the study specific prevalence of disease *π*_*i*_ and the *S*_*ij*_ and *C*_*ij*_ of the *J*_*i*_ tests used in study *i*, which is a subset of the *J* different tests used across the *I* studies of the meta-analysis.

##### *Model 4: Hierarchical Latent Class Model*

In essence, the most basic hierarchical latent class model (Model 4) is constructed through a combination of equations 1 and 5. While previously the reference test was presumed to be 100 % sensitive and specific, in Model 4 all *S*_*ij*_ and *C*_*ij*_, including those of the reference tests, are modeled using separate bivariate normal distributions as in Equation 1. The observed data is assumed to come from the multinomial distribution described in Equation 5. Again, like Model 1, this model ignores the correlation among test results from the same study. The prevalences *π*_*i*_ can be assumed to be different for each study or to have a common normal distribution, $\pi _{i} \sim N\left (\mu _{\pi },\sigma ^{2}_{\pi }\right)$.

##### *Model 5: Network-based Hierarchical Latent Class Model*

By rewriting the *θ*_*Sij*_ and *θ*_*Cij*_ in terms of *μ*_*Si*_,*μ*_*Ci*_,*δ*_*Sij*_, and *δ*_*Cij*_ as in Eq 4, we can again take into account study level effects. The hierarchical modeling is equal to Model 3, the only difference is at the study level as described in Eq 5. This model thus adjusts the meta-analysis for the use of imperfect reference tests. By using the expanded Smith −*Spiegelhalter*− Thomas model of Lu et al. [14] at the second level of the hierarchy, study level effects are eliminated without the need to limit the analysis to direct comparisons only.

### Model estimation and prior specification

Models are estimated in a Bayesian framework using Markov Chain Monte Carlo (MCMC) methods with OpenBUGS 3.0.3 called from within R 3.0.1 using the BRugs library. The Bayesian approach allows the estimation of complex, joint models and the combination of prior information, e.g. on the value of the reference test used, in the meta-analysis of new diagnostic tests. To complete the Bayesian model, priors need to be provided for all model parameters. OpenBUGS code for the models and full specifications of the priors are in Additional file 2. Convergence was checked using visual inspection of trace plots of the Markov chains and the Gelman-Rubin diagnostic statistic [22].

For parameters related to the index tests of interest, we consider it generally most appropriate to use uninformative priors. Specifically, we used normal priors with mean *μ* equal to zero and standard deviation *σ* equal to 1.69 for logit-transformed probabilities. This prior matches a uniform prior over the interval [0,1] in the first two moments on the probability scale [23]. When appropriate, these priors were bounded to avoid label switching [20]. Label switching is a problem arising in MCMC estimation of latent class models when two equivalent solutions are possible which give rise to identical observed data [24, 25]. The problem can be avoided by constraining *S* or *C* of one or more test to be ≥0.5. For the contrast in *S* and *C*, expressed as log ORs, normal priors with *μ*=0 and a large standard deviation, e.g. *σ*=10 can be used. For the variance-covariance matrices, we construct non-informative priors using uniform priors for standard deviations and correlations.

The model was specified using a logit link function and results are estimated on the log-odds scale. The MCMC approach as implemented in OpenBUGS allows to obtain posterior distributions of all functions of the estimated parameters, as the average *S* and *C* of the index tests and differences between *S* and *C* of the different tests. We illustrate this in the OpenBUGS code in Additional file 2. We used the 2.5 and 97.5 th percentiles of the sampled posterior distribution of the statistics of interest as bounds for the 95 % credible intervals.

If we want to use information from previous phases of the research, we can use informative priors. It may for example be appropriate to use information obtained from a previous meta-analysis of case-control studies when performing a meta-analysis of phase IV studies, i.e. studies recruiting clinically suspect patients consecutively in a representative clinical setting [7]. However, given that the phase IV design ensures the most realistic assessment of the performance of a test when used as a diagnostic tool in the target population [6], we may want to reduce the influence from these prior phases by using a prior which is more diffuse than the actual results from the prior meta-analysis. In the latent class model Model 4, we can use informative priors for the diagnostic accuracy of the reference test. It is likely that some information on the accuracy of the reference tests is available. In fact, standard analysis assumes *S* and *C* of the reference test to be 100 %, which can be considered to be very strong deterministic prior from a Bayesian viewpoint [26]. Priors for the accuracy of reference test can be obtained from the literature or expert opinion [10].

### Simulation study

To assess the performance of the different models and to uncover possible bias of combining data without proper control for study specific effect or adjustment for the use of imperfect reference standards, we performed a simulation study using two different scenarios. For each scenario, we generated 250 sets of 20 diagnostic studies. We analyzed each simulated data set using the models described above using the logit for the link function *g*(.). We evaluated the models using coverage probabilities (the proportion of replications in which the 95 % credible interval contained the true value) and power (the proportion of replications in which a difference in *S* and *C* between the two tests of interest was detected). In Scenario 1, we simulated a setting without systematic bias but where a common imperfect reference test is used to assess the diagnostic accuracy of the index tests in all primary studies. In Scenario 2, we simulated the situation of two index tests which are assessed in primary studies that tend to use different reference standards. This situation may rarely occur in practice, but was selected to assess how the model performed in an extreme situation with systematic bias due to imperfect reference tests. A full description of the simulation study setup is in Additional file 3.

### Real data example

**Fig. 1:**
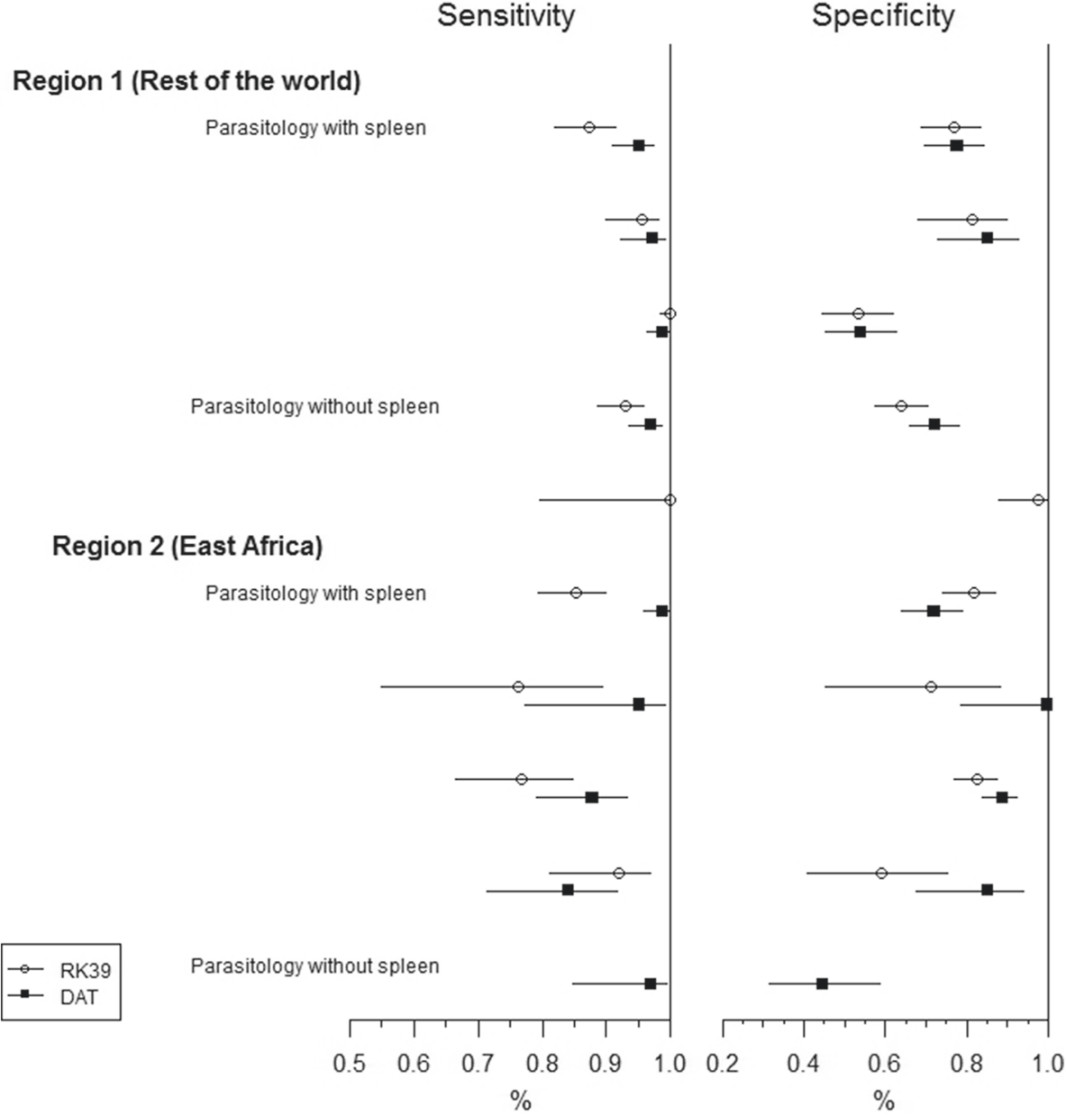
Forest plot for real data example. Estimated sensitivity and specificity of the RK39 dipstick (open circles) and DAT (closed squares) with 95 % confidence interval, using parasitology as gold standard

We applied the models to data obtained in a meta-analysis of rapid diagnostic tests for visceral leishmaniasis, which we described earlier [10, 27]. In the published meta-analysis, the focus was on estimating the diagnostic accuracy of individual tests. We extracted the data relevant for the comparison of one rapid diagnostic test, the RK39 dipstick, with that of the direct agglutination test (DAT) as a test case for the application of the methods developed in the current paper. We limited the data for this test case to primary studies that included the RK39 dipstick or DAT with at least one other index test and microscopical examination as a reference test for which full data was available in the published primary study. We selected all index tests which were used in more than one study. In total, we included 10 primary studies, four index tests (DAT, RK39-dipstick, IFAT, KAtex) and two reference tests (parasitology including spleen aspirate, parasitology not including spleen aspirate) (Table 3). All tests are specific to VL and consequently can be expected to related to the same underlying latent variable. The data are shown in Fig. 1 and Appendix 4. Note that the current study is used as “proof-of-concept” of the statistical modeling approach and not as a complete meta-analytic comparison of the two tests which would require a more extended search strategy.

**Table 3:** Overview of the real data example: a comparative meta-analysis of the RK39 dipstick and direct agglutination test (DAT) for the diagnosis of visceral leishmaniasis. The total sample size (N) and availability of test results (X) is given for all 10 studies. Other tests: IFAT=indirect fluorescent antibody test, KAtex =latex agglutination test, spleen =parasitological examination of tissue aspirates including spleen sample, no spleen: parasitological examination of tissue aspirates not including spleen sample

Study information		Index tests				Reference test		
Publication	Country	RK39	DAT	KAtex	IFAT	Spleen	No Spleen	N
Boelaert-1999	Sudan		X		X		X	59
Boelaert-2004	Nepal	X	X		X	X		309
Boelaert-2008	Nepal	X	X	X		X		158
Boelaert-2008	India	X	X	X		X		352
Boelaert-2008	Kenya	X	X	X		X		307
Boelaert-2008	Ethiopia	X	X	X		X		35
Boelaert-2008	Sudan	X	X	X		X		291
de Assis-2012	Brazil	X	X		X		X	407
Toz-2004	Turkey	X			X		X	42
Veeken-2003	Sudan	X	X			X		77

The aim of this modeling exercise was to estimate the differences in *S* and *C* between the RK39-dipstick and DAT. A previous meta-analysis indicated that the diagnostic accuracy of the RK39, and possibly also of the DAT, may be lower in East-Africa compared to other geographic regions [28]. To correct for these differences, we included a fixed region effect (East-Africa vs. rest of the world) for *S*. We fitted the five models listed in Table 2. In the previous study [10], we obtained expert opinion on the diagnostic accuracy of the two reference tests. Expert opinion on the diagnostic accuracy of parasitology including spleen aspirate varied between 88 and 95 % for *S* and between 95 and 100 % for *C*. For parasitology without spleen aspirate, expert opinion varied for *S* between 70 and 80 % and between 95 and 100 % for *C*. We used this information to determine the priors in estimation of the models allowing for imperfect reference standards.

## Results

### Measures of relative value of diagnostic tests

The results of our simulation study indicated that in a realistic setting, bias in estimating the difference in *S* and *C* between two index tests due to the use of an imperfect reference standard can be relatively limited (Additional file 1). Strong bias only occurred if the errors of one index test were strongly correlated with those of the reference test while the errors of the second index test were uncorrelated with those of the reference test. Similar observations can be made for the relative *S* and *C*. When the comparisons were expressed as odds-ratios or when using the relative Diagnostic Odds Ratio as a summary statistics, bias was more substantial and occurred even with uncorrelated errors. This corresponds to the findings from Zhang et al. who report that also in the meta-analysis of RCTs the odds-ratio is not always a suitable summary statistic [5].

### Model performance: simulation study

**Fig. 2:**
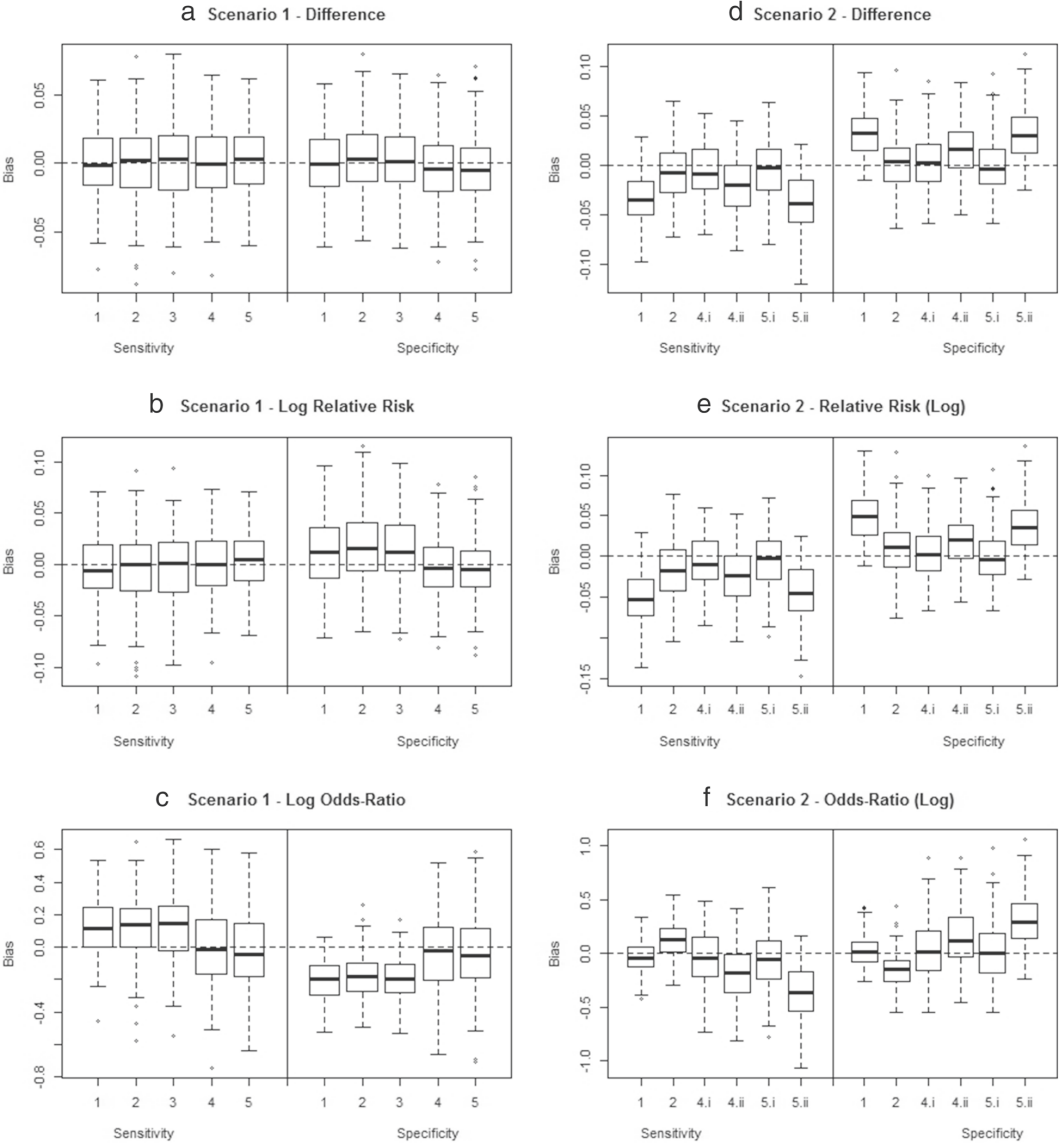
Summary of simulation results. Bias in estimates of the contrasts in diagnostic accuracy from the proposed meta-analytical models applied in the simulation study. The boxplots present the bias in $\hat {S}_{D12}$ and $\hat {C}_{D12}$ (first row), $\hat {S}_{RR12}$ and $\hat {C}_{RR12}$ (second row), $\hat {S}_{OR12}$ and $\hat {C}_{OR12}$ (third row). The first column presents Scenario 1 where a common imperfect reference standard with moderate *S* and high *C* was used, the second scenario 2 where systematic bias is induced by differing reference standards. Full explanation of the model is in the text; full explanation of the simulation setup and results in Additional file 3. Note for Scenario 1: For models 1 to 3 disease status was estimated from the results of *T*_4_. Note for Scenario 2: Models 4 and 5 were applied both assuming it is known that the reference tests differ across studies (4a and 5a) and ignoring the difference in reference tests (4b and 5b)

Results of the simulation study of the model performance are described in detail in Additional file 3. The bias in estimating the contrasts in *S* and *C* between *T*_1_ and *T*_2_, expressed as a difference, relative risk or odds-ratio is summarized in Fig. 2.

In Scenario 1, where a common imperfect reference standard with moderate *S* and high *C* was used, a naive analysis assuming the reference test was perfect (Models 1–3), resulted in bias in estimating the odds-ratios (Fig. 2c) and to a lesser extent also the relative risks (Fig. 2b). If the contrast of interest was expressed as a difference (Fig. 2a), a true gold standard was available (Fig. 2a–c, Models 1–3), or if a latent class model was used to allow for imperfect reference tests (Fig. 2a–c, Models 4 and 5), no bias was apparent. Allowing for imperfect reference tests resulted in a lower power compared to the situation that a perfect reference test was available (Additional file 3, Table 3).

In Scenario 2, the reference standard of the simulated studies varied according to the index tests studies, which could result in systematic bias. In the analysis of Scenario 2, Models 4.i and 5.i correspond to the situation where the researchers knew of the differences in reference standard used across studies and that the variation in reference standard was thus a known source of bias. Models 4.ii and 5.ii correspond to the situation that researchers are unaware of the differences in reference standards among studies and that consequently the variation in reference standard was an unknown source of bias. This may occur if researchers do not provide sufficient detail in the primary publications on the exact modalities of the reference test procedures. For example, in the diagnosis of VL microscopical examination of spleen aspirates is the preferred reference test, while bone marrow aspirates show a limited *S*. Often researchers indicate their reference test to be based on spleen aspiration. However in closer assessment of these publications, it can become apparent that some researchers perform spleen aspiration on nearly all subjects while others may preform spleen aspiration on only a minority of subjects. Ignoring these difference in reference tests may lead to bias. Incorrectly assuming the reference test were perfect resulted in substantial bias, especially when ignore study level effects (Fig. 2d–f, Model 1). When correcting for the use of imperfect reference tests using LCA (Models 4.i and 5.i), unbiased estimates for the differences in diagnostic accuracy between *T*_1_ and *T*_2_ were obtained (Fig. 2d–f, Models 4.i and 5.i). If the data were however analyzed ignoring the differences between the reference tests, the differences in diagnostic accuracy between *T*_1_ and *T*_2_ were overestimated (Fig. 2d–f, Models 4.ii and 5.ii).

### Real data example: diagnostic tests for visceral leishmaniasis

Results of modeling of the VL data are in Table 4. All models indicated that *S* of DAT (*S*_2_) was 8 to 11 % higher, compared to the *S* of RK39 (*S*_1_), in East-Africa, but this difference did not reach statistical significance. In the rest of the world, estimates of *S*_1_ and *S*_2_ were similar. Differing modeling strategies or allowing for imperfect reference standards did not impact estimates of *S* or comparisons of *S* between the two index tests of interest. This is as expected as the parasitological reference tests show a similar and high *C*.

**Table 4:** Results of the meta-analysis of the diagnostic tests for visceral leishmaniasis

	Parasitology as Gold Standard			No Gold Standard	
	Model 1	Model 2	Model 3	Model 4	Model 5
Parameter	Estimate	Estimate	Estimate	Estimate	Estimate
*S*_1_ (R1)	94.2	95.5	94.8	95.9	94.7
*S*_1_ (R2)	85.2	84.0	86.5	84.6	88.1
*S*_2_ (R1)	95.7	97.2	96.4	96.3	96.4
*S*_2_ (R2)	94.4	93.5	95.4	96.1	96.5
*C*_1_	78.6	75.5	78.3	90.2	91.0
*C*_2_	80.1	80.9	81.5	93.0	94.1
*S*_*D*12_ (R1)	1.5	1.7	1.6	0.4	1.7
*S*_*D*12_ (R2)	9.2	9.6	8.9	11.5	8.4
*C*_*D*12_	1.5	5.3	3.2	2.8	3.1
*S*_*RR*12_ (R1)	1.02	1.02	1.02	1.00	1.02
*S*_*RR*12_ (R2)	1.11	1.12	1.10	1.14	1.10
*C*_*RR*12_	1.02	1.07	1.04	1.03	1.03
*S*_*OR*12_ (R1)	1.3	1.7	1.5	1.1	1.6
*S*_*OR*12_ (R2)	2.7	2.8	3.3	4.7	3.9
*C*_*OR*12_	1.1	1.4	1.2	1.5	1.7

*S*_*i*_ and *C*_*i*_: sensitivity and specificity of Test *i*; *S*_*D*12_ and *D*_*D*12_: difference in sensitivity and specificity between Test 1 and Test 2; *S*_*RR*12_ and *D*_*RR*12_: relative sensitivity and specificity of Test 1 compared to Test 2 as a relative risk; *S*_*OR*12_ and *D*_*OR*12_: relative sensitivity and specificity of Test 1 compared to Test 2 expressed as an odds-ratio. R1 and R2 indicate estimates obtained for East-Africa and the rest of the world, respectively

In contrast, allowing for imperfect reference tests (models 4 and 5) resulted in considerably higher estimates for *C* of both the RK39 dipstick and DAT compared to models assuming perfect reference tests were used (models 1–3). False negative results for the reference tests may have resulted in reduced estimates of *C*_1_ (75.5–78.6 %) and of *C*_2_ (80.1–81.5 %). Allowing for imperfect reference standards resulted in considerable higher estimates for *C*_1_ (90.2–91.0 %) and *C*_2_ (93.0–94.1 %).

In the analyses that used parasitology as a, presumed perfect, reference test, a substantial difference between *C*_1_ and *C*_2_ ($\hat {C_{2}} - \hat {C_{1}}$ = 5.3 %) was observed when limiting the analysis to direct comparisons only (Model 2). On the other hand, Model 1, based on independent estimation of *C*_1_ and *C*_2_, showed a much smaller difference ($\hat {C_{2}} - \hat {C_{1}}$ = 1.5 %). The model using direct and indirect comparisons (Model 3) showed intermediate results (3.2 %). This can be explained by the fact that the studies in which no direct comparison was possible between the RK39 dipstick and DAT showed contradictory results to the studies with direct comparisons. These studies also used the least sensitive reference standard which may explain that results of Models 4 and 5, both allowing for imperfect reference standards, were similar.

## Discussion

In this paper, we developed a novel model to perform a comparative meta-analysis of the accuracy of two or more diagnostic tests when a perfect reference standard is unavailable. In a first step, we assessed the bias of comparative measures of the diagnostic accuracy of two tests induced by the use of an imperfect reference test. We observed that the difference in *S* and *C* may be the least subject to bias while at the same time being easily understandable to users of the meta-analysis results. In our modeling approach, we combined LCA with models developed for the mixed treatment comparisons meta-analysis of RCTs. The modeling framework accommodates a broad range of studies, including “Multiple Test Comparison”, “Randomized Test Comparison”, and “Between-Study Test Comparison” studies according to the terminology of Takwoingi et al., with the first two designs offering the most robust comparative data [3]. In a simulation study, the resulting model showed adequate performance, even if some aspects of the data generating mechanism were ignored. The simulation study also stressed the importance of accurate and complete extraction of the data from the primary studies when performing a DTA review. When differences in reference tests were ignored, biased estimates of the relative accuracy of the competing tests were unavoidable. This highlights the importance of complete and transparent reporting of DTA studies as promoted by the STARD initiative [29]. For a correct analysis of the data, the index and reference tests should be accurately described. Any cut-offs used to classify test results as positive or negative should also be reported and results for all subjects should be given, including subjects with incomplete or equivocal test results. The cross-classification of all test results should be presented in a format similar to that of the motivating example dataset in Additional file 4. The fact that meta-analysis is possible using imperfect reference tests suggests it may be more efficient to design future studies with multiple imperfect tests rather than using a single “as-accurate-as-possible” reference test, as has been shown in the analysis of epidemiological studies with imperfect measures of exposure [30, 31]. When applied to a dataset on visceral leishmaniasis diagnostic tests, the model indicated that *C* of the two tests of interest may have been underestimated due to the use of imperfect reference test. Our novel modeling approach, combining latent class analysis with hierarchical meta-analysis modeling, allowed the estimation of the difference in accuracy of the two index tests without making strong assumptions on the performance of the reference tests used. However, as in all meta-analyses, care should be taken that the studies combined are in fact comparable. While our approach corrects for bias and heterogeneity induced by the use of imperfect reference tests, other types of bias as publication and spectrum bias, can result in incorrect meta-analysis results. The approach can be combined with meta-regression techniques to reduce heterogeneity.

As limitations of our approach the following points can be given, which can indicate future avenues for further progress in this field. As a first limitation, we chose to compare diagnostic tests based on the sensitivity and specificity, and in particular based on the difference in these quantities among competing tests. Focusing on differences in *S* and *C* leads to results which are easily understandable for potential users. However, a test can be superior to another with respect to *S* while inferior with respect to *C*. In this case, selecting the optimal test can be difficult. Using a single summary measure of diagnostic accuracy, as the relative diagnostic odds-ratio (rDOR) can make comparisons among tests easier [32]. Theoretically, the test with the highest DOR may be preferred. However, this may not always be the case as the potential risk of a false positive result may be different from the risk of a false negative result. It may be easier for users to balance an increase in *S* versus decreases in *C*. In addition, the rDOR may be more prone to bias as we have shown for the OR difference in *S* and *C*. In our model formulation, the rDOR can be easily obtained. If the primary parameter of interest is however the rDOR, an alternative model formulation, for example an extension of the hierarchical summary ROC model [9, 33], may be more appropriate.

To allow estimation of the model, we made considerable simplifications to the variance-covariance structure of our parameter space. Not all these simplification may be warranted and a more general variance-covariance structure may refine estimates from this model. Fitting a general variance-covariance matrix however results in important computational difficulties. Our simulation study indicated that these limitations do not necessarily invalidate analysis results, but further research is needed to assess when this may no longer be the case. Modeling the variance-covariance matrix via partial autocorrelations [15] may allow the fitting of more complex model. We accommodated study effects using contrast-based (CB) approaches. However, in RCT arm-based approaches that correctly incorporate correlations have been shown to be superior to CB methods [5]. Further development of the equivalent models for DTA reviews, Models 1 and 4 in our setting, incorporating the correlations induced by study levels, is needed. Model 4 which corrects for imperfect reference tests, but ignores study effects, performed well in our simulation study. However, it is vulnerable to bias from study-specific effects. the model would need extensions to incorporate dependencies between test results from the same study, as for example was done for the meta-analysis of RCTs in Zhang et al. 2014 [5], before it is recommended as a general method for the meta-analysis of DTA studies above Model 5. However, if the accuracy of the different index tests is not strongly correlated across studies, Model 4 may perform equally well as Model 5 and may offer advantages in terms of identifiability and computational feasibility.

We showed how prior information, e.g. on the diagnostic accuracy of the reference test, can be used to aid model estimation in the case of the hierarchical latent class model. This is in line with the methods we have earlier developed for the meta-analysis of the diagnostic accuracy of a single test when using an imperfect reference standard [10]. In the case of the network based latent class model, it is however much less clear how this information can be used. The diagnostic accuracy of each test is in this model a linear combination of an overall, study-specific, probability of testing positive on all tests and a number of contrasts in diagnostic accuracy among these tests. More research is needed on how priors can be constructed for this model, e.g. using the priors for conditional probabilities rather than for *S* and *C* directly [26].

DTA studies can be expected to exhibit considerable heterogeneity and may be more prone to bias and inconsistency between direct and indirect comparisons compared to RCTs. Applications of network meta-analytic model to DTA studies must be performed with care and further development of statistical methods are needed. The literature on network-based meta-analysis of RCTs contains many additional tools, for example to assess consistency of estimates obtained from direct versus indirect comparisons [34–36], assess heterogeneity among studies [37], detect outlying studies [38] and correct for bias [39]. We only performed a limited application of techniques developed in this context. Expanding these techniques to DTA meta-analyses may be a valuable direction of research. In particular, it is important to expand the concept of consistency of comparisons across networks to the context of DTA reviews [40, 41].

Alternative approaches to the comparative meta-analysis of diagnostic tests are proposed. The regression approach of Macaskill et al. [9] can be seen as a variation of our model 2 in which the relative *S* and *C*, expressed as an odds-ratio, between tests is constant across studies. For the case all tests are applied to all subjects, Trikalinos at al. [42] describe a model which fully accounts for the within-study correlation between the tests’ subject-specific S and C. This approach can be more efficient than the methods proposed in the current manuscript. However, both approaches need further empirical and simulation studies to assess their relative merits. Different models may be most appropriate depending on the application. In case it is suspected that reference tests may show only limited *S* or *C*, the analysis method should allow for the use of imperfect reference test. If important study-level effects are expected, proper control for confounding by these effects is needed. If there is important uncertainty on the value of the reference test or the presence of study level effects, it will be preferable to fit several models and assess the robustness of the results to the assumptions. At this stage of research, it is not possible to provide a general recommendation on the optimal modeling approach for the meta-analysis of comparative DTA reviews.

## Conclusions

The models developed in this paper are promising and can improve the comparison of the diagnostic accuracy of competing tests in DTA systematic review. This is however only true if the studies and especially information on the reference tests used are described in sufficient detail. If the reporting of the studies does not provide sufficient detail, it may be better to limit the meta-analysis to direct comparisons. Further work refining the modeling approach, especially with respect to the specification of more general covariance structures and the use of measures of consistency of direct versus indirect comparisons, can further improve these methods.

## Additional files

Additional file 1
**Simulation Study for the Selection of Appropriate Statistics for a Comparative DTA Review.** (PDF 1208 KB)

Additional file 2
**Software Code, Prior Specification, and Possible Structures for Variance-Covariance Matrices.** (PDF 149 KB)

Additional file 3
**Simulation Study of the Modeling Approach.** (PDF 491 KB)

Additional file 4
**Visceral Leishmaniasis Data.** (PDF 44 KB)
