# Supplementary material for: A general framework for comparative Bayesian meta-analysis of diagnostic studies
Source: BMC Med Res Methodol. 2015 Aug 28;15:70. doi: 10.1186/s12874-015-0061-7 (PMC4552463; doi:10.1186/s12874-015-0061-7)
Supplement: Additional file 1 — Simulation Study for the Selection of Appropriate Statistics for a Comparative DTA Review. (PDF 1208 KB) [file 12874_2015_61_MOESM1_ESM.pdf]

Additional file 1 — Simulation Study for the Selection of Appropriate Statistics for a Comparative DTA Review

1.1: Definitions

Basic statistics to describe the accuracy of a diagnostic test are the sensitivity $S = P(T+|D+)$, where $T+$ is a positive test result and $D+$ indicates subjects with the disease of interest, and the specificity $C = P(T-|D-)$, where $T-$ is a negative test result and $D-$ indicates subjects free of the disease of interest. S and C can be compared between two tests T_1 and T_2 using:

1 the difference in S ($S_{D12} = S_1 - S_2$) and C ($C_{D12} = C_1 - C_2$),
2 the relative S ($S_{RR12} = S_1/S_2$) and C ($C_{RR12} = C_1/C_2$),
3 the odds-ratios $S_{OR12} = [S_1 \times (1 - S_2)]/[(1 - S_1) \times S_2]$ and $C_{OR12} = [C_1 \times (1 - C_2)]/[(1 - C_1) \times C_2]$.

The relative diagnostic odds ratio $rDOR_{12} = [S_1 \times C_1 \times (1 - S_2) \times (1 - C_2)]/[S_2 \times C_2 \times (1 - S_1) \times (1 - C_1)]$ summarizes the relative diagnostic accuracy of two tests in a single number.

1.2: Objectives

The aim of this simulation study is to determine to which extent the different possible comparison statistics, as defined in 1.1, are affected by the use of a imperfect reference standard. The bias induced by using an imperfect reference test in estimating S and C are well described in the literature [7,8]. How this affects the comparison of the accuracy of diagnostic tests is less studied.

In this simulation study, we describe the situation of a single diagnostic accuracy study, in which 3 test are performed. The first tests are index tests that we want to compare. The third test is a reference test which is used to estimate the S and C of the two index tests. The S and C of the index tests are estimated through comparison with the imperfect reference test. From these estimated, or apparent, S and C, we calculate the contrasts described in 1.1.

We compare these apparent summary statistics of the relative accuracy (difference, relative S and C, and odds-ratios) with those calculated when the true disease status would have been known. This, we compare them with the true underlying population values of these statistics. We assess only the bias induced by the use of imperfect reference test and did not consider in our simulation other characteristics, for example variability, of the summary statistics. The standard error of the summary statistics will also depend on the estimation method used and is considered in the simulation study of the different models (Additional File 3).

1.3: Simulation Study Setup

For our simulation study, we selected a typical situation as is commonly encountered in infectious diseases research. In this scenario, our aim is to compare two index tests T_1 and T_2 with S_1=90%, S_2=85%, C_1=85%, and C_2=90%, i.e. one test T_1 is more sensitive, while the other test T_2 is more specific. The simulated (true) values for the contrasts between T_1 and T_2 are consequently 5.0% for S_{D12}, -5.0% for C_{D12}, 1.06 for S_{RR12}, 0.94 for C_{RR12}, 1.58 for S_{OR12}, and 0.63 for C_{OR12}. The $rDOR_{12}$ is equal to 1. The data were generated using dependent binomial distributions in R.

The dependency of between the distributions were induced by using random effects on the logit probability scale.

To assess the influence of an imperfect reference test in estimating the relative diagnostic accuracy of two index tests, we estimated this diagnostic accuracy through comparison with test T_3. T_3 has S_3=80% and C_3=95%, i.e. it is a test which is highly specific but has lower sensitivity. This would commonly be encountered with techniques which rely on the identification of infectious agents through microscopy or culture.

In the simulation, we varied the correlation between the two index tests and the reference test from a limited negative correlation to a strong positive correlation. The correlation was summarized as the log OR of a positive result on the index test dependent on the result of the reference test. We simulated 250 datasets of 10,000 subjects with a prevalence of 50% and calculated the relative accuracy of T_1 and T_2 with the statistics described above. We selected 250 simulations to obtain sufficient precision, while not requiring excessive calculation time. As the aim of the simulation study to assess the bias induced by the use of imperfect reference standards, a large sample size (10,000) for the individual simulation runs was selected so that the sampling error plays no role in the simulation. A prevalence of 50% was chosen in the simulation study, as this offers the most information and provides an equal comparison of the amount of false positives and false negatives. Results were comparable when selecting other prevalences (10%, 90%, data not shown).

1.4: Results

Results of calculating the relative diagnostic accuracy of T_1 and T_2, through comparison with T_3 are summarized in Figure 1 to 4.

Sensitivity S and Specificity C

Looking at the estimates of the diagnostic accuracy of index tests T_1 and T_2, we observed that the bias in estimating S and C depended on the correlation, conditional on the diseases status, between the index test and the imperfect reference test T_3. If the errors of T_1 and T_3 were uncorrelated, then the estimates S and C of T_1 were $\hat{S}_1 = 86\%$ and $\hat{C}_1 = 72\%$. If however false positive and false negative results for T_1 and T_3 were strongly positively correlated (log OR = 1.1) then $\hat{S}_1 = 92\%$ and $\hat{C}_1 = 77\%$. If they were negatively correlated (log OR = -0.4) then $\hat{S}_1 = 84\%$ and $\hat{C}_1 = 71\%$. Similar results were obtained for T_2. These results correspond what is already well reported in the literature.

Differences S_{D12} and C_{D12}

Figure 1 shows the bias in the difference S_{D12} for different correlations between T_1 and T_3 (X-axis) and T_2 and T_3 (Y-axis). In Figure 1.a false positive errors are uncorrelated, i.e. in the non-diseased T_1, T_2 and T_3 are independent, but the false negative errors, i.e. results in the diseased subjects, are positively or negatively correlated. If the correlations between T_1 and T_3 and between T_2 and T_3 were equal, then the estimate of S_{D12} was approximately unbiased. If T_1 was more strongly correlated to T_3 than T_2, there was a positive bias $(\hat{S}_{D12} - \bar{S}_{D12}) > 0$. For example, if false negative results for T_1 were strongly positively correlated with those of the

reference test while errors for T_2 were uncorrelated, the estimated difference $\hat{S}_{D12}$ was equal to 10%, a bias of +5%. Vice versa, if T_2 was more strongly correlated to T_3 than T_1, there was a negative bias $(\hat{S}_{D12} - \bar{S}_{D12}) < 0$. Returning to our example, if false negative results for T_2 were strongly positively correlated with those of the reference test while errors for T_1 were uncorrelated, the estimated difference $\hat{S}_{D12}$ was equal to 0%, a bias of −5%. The same trends were seen when the false negative results were uncorrelated, but the correlation of the false positives was allowed to vary. These effects can be expected a priori. If the errors of index and reference tests are positively correlated, S and C will be relatively over-estimated (or less under-estimated) compare to the case the errors are not correlated.

Relative risks S_{RR12} and C_{RR12}

Similar results were obtained for the contrast expressed as a relative risk (Figure 2). If false negative results for T_1 were strongly positively correlated with those of the reference test while errors for T_2 were uncorrelated, the estimated relative risk $\hat{S}_{RR12}$ was equal to 1.12, a bias of +6%. However if false negative results for T_2 were strongly positively correlated with those of the reference test while errors for T_1 were uncorrelated, the estimated relative risk $\hat{S}_{RR12}$ was equal to 0.98%, a bias of −8%.

Odds-ratios S_{OR12} and C_{OR12}

Expressed as an odds-ratio, the bias was stronger (Figure 3) and occurred even with uncorrelated errors. If results for T_1, T_2, and T_3 were conditionally independent, the estimated OR $\hat{S}_{OR12}$ was equal to 1.42, or a bias of −12% compared to the true value of 1.58. If false negative results for T_1 were strongly positively correlated with those of the reference test while errors for T_2 were uncorrelated, the estimated OR $\hat{S}_{OR12}$ was equal to 2.26, a bias of +42%. If false negative results for T_2 were strongly positively correlated with those of the reference test while errors for T_1 were uncorrelated, the estimated OR $\hat{S}_{OR12}$ was equal to 0.83, a bias of −48%.

The relative diagnostic odds ratio $rDOR_{12}$

Lastly, the relative Diagnostic Odds-Ratio showed a similar bias as S_{OR12} and C_{OR12} (Figure 4). If results for T_1, T_2, and T_3 were conditionally independent, the estimated dDOR $r\hat{DOR}_{12}$ was equal to 1.09, or a bias of 9% compared to the true value of 1. If false negative results for T_1 were strongly positively correlated with those of the reference test while errors for T_2 were uncorrelated, the estimated rDOR $r\hat{DOR}_{12}$ was equal to 2.15, a bias of +115%. If false negative results for T_2 were strongly positively correlated with those of the reference test while errors for T_1 were uncorrelated, the estimated estimated rDOR $r\hat{DOR}_{12}$ was equal to 0.48, a bias of −52%.

1.4: Figures

Figure 1 Bias (%) in the estimation of the difference in S between two tests ($\hat{S}_{D12}$) for different correlations between T_1 and T_3 (X-axis) and T_2 and T_3 (Y-axis).

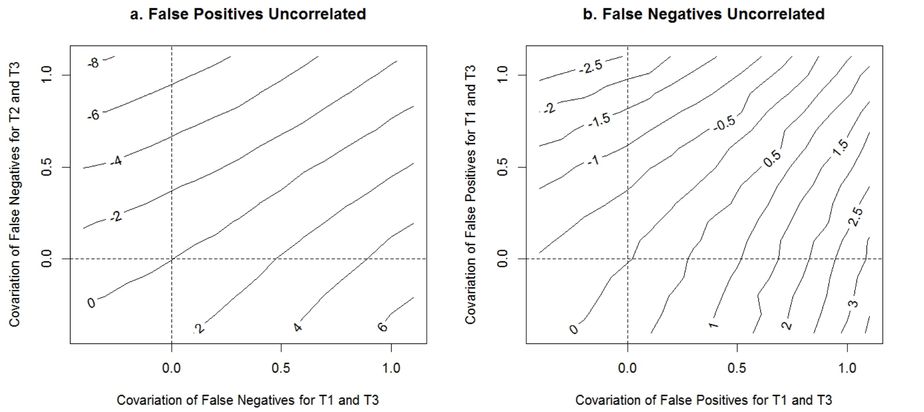

Notes: The correlation is summarized as the log OR of a positive result on the index test dependent on the result of the reference test. Contours indicate the bias in percentage points. Dotted lines indicate 0 correlation.

Figure 2 Bias (%) in the estimation of the relative risk in S between two tests ($\hat{S}_{RR12}$) for different correlations between T_1 and T_3 (X-axis) and T_2 and T_3 (Y-axis).

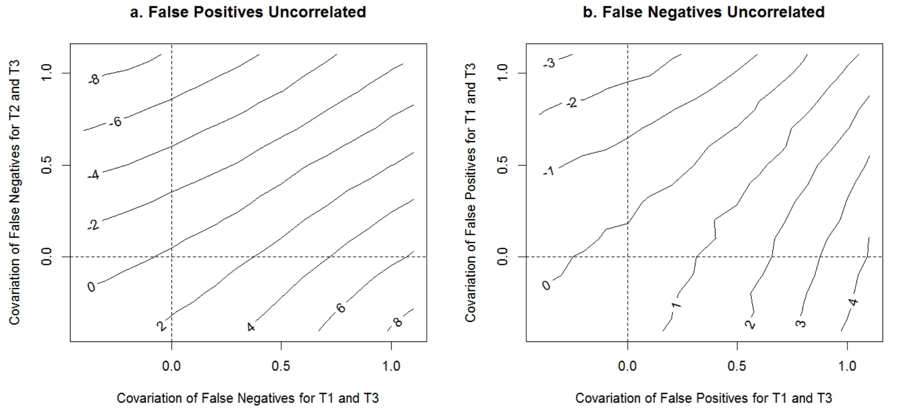

Notes: The correlation is summarized as the log OR of a positive result on the index test dependent on the result of the reference test. Contours indicate the bias in percentage points. Dotted lines indicate 0 correlation.

Figure 3 Bias (%) in the estimation of the odds ratio in S between two tests ($\hat{S}_{OR12}$) for different correlations between T_1 and T_3 (X-axis) and T_2 and T_3 (Y-axis).

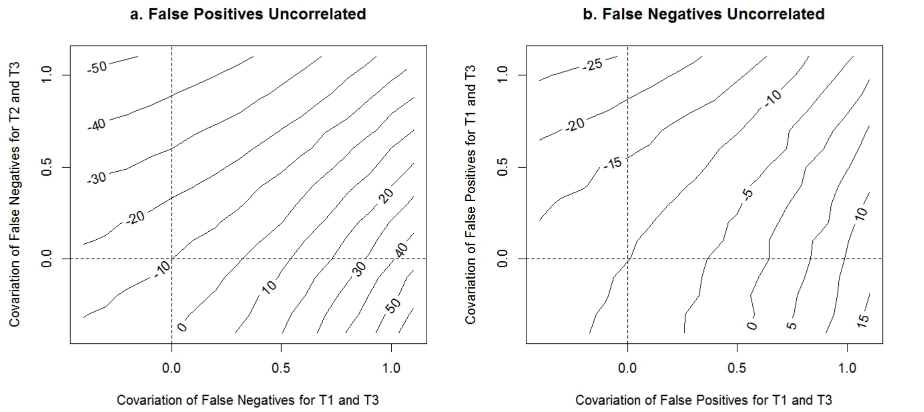

Notes: The correlation is summarized as the log OR of a positive result on the index test dependent on the result of the reference test. Contours indicate the bias in percentage points. Dotted lines indicate 0 correlation.

Figure 4 Bias (%) in the estimation of the relative diagnostic odds ratio between two tests ($r\hat{DOR}_{12}$) for different correlations between T_1 and T_3 (X-axis) and T_2 and T_3 (Y-axis).

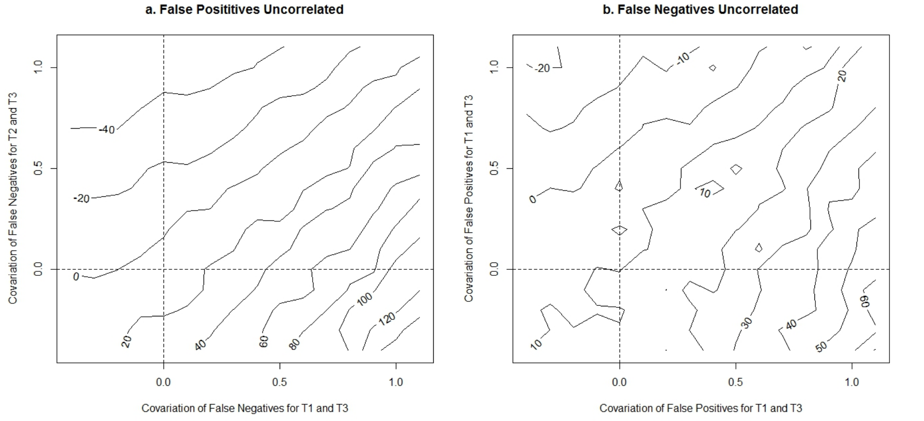

Notes: The correlation is summarized as the log OR of a positive result on the index test dependent on the result of the reference test. Contours indicate the bias in percentage points. Dotted lines indicate 0 correlation.
