# Supplementary material for: A general framework for comparative Bayesian meta-analysis of diagnostic studies
Source: BMC Med Res Methodol. 2015 Aug 28;15:70. doi: 10.1186/s12874-015-0061-7 (PMC4552463; doi:10.1186/s12874-015-0061-7)
Supplement: Additional file 2 — Software Code, Prior Specification, and Possible Structures for Variance-Covariance Matrices. (PDF 149 KB) [file 12874_2015_61_MOESM2_ESM.pdf]

Additional file 2 — Software Code, Prior Specification, and Possible Structures for Variance-Covariance Matrices

Note: in the example code the index i indicates the subject level, j the study level and k the individual tests.

2.1: Example code for Model 1

```
model{
  ### Basic model for results ###
  # The total nr. of patients is specified in the data as variable nPats
  for (i in 1:nPats){
    # Disease status is provided in the data through the gold standard
    # result GS which is a vector of length nPats with
    # 1=diseased and 0=non-diseased
    status[i] <- GS[i]
    # Results for each test follow independent Bernoulli distributions,
    # variable Y[i=patient nr, k=test nr], with probability determined
    # by the study-specific, logit-transformed S and C
    for(k in 1:2){
      Y[i,k] ~ dbern(P[i,k])
      }
    logit(P[i,1]) <- status[i] * alpha1[study[i],1] +
        (1-status[i]) * alpha1[study[i],2]
    logit(P[i,2]) <- status[i] * alpha2[study[i],1] +
        (1-status[i]) * alpha2[study[i],2]
    }
  ### Model for study level results ###
  for(j in 1:nStudy){
    # Model for study-specific, logit-transformed S (alpha) and C (beta)
    alpha1[j,1:2] ~ dmnorm(mu1[],R1[,])
    alpha2[j,1:2] ~ dmnorm(mu2[],R2[,])
    }
  ### Priors for study level results ###
  # Priors for average (logit) probability of testing positive:
  # vague priors that are equivalent to uniform over [0,1] interval
  # Note that 0.35=1/1.69**2
  # Constraints on mu1 and mu2 to avoid label switching
  # - mu1[1] and mu2[1] constrained to be positive: average S > 0.5
  # - mu1[2] and mu2[2] constrained to be negative: average 1-C < 0.5
  mu1[1] ~ dnorm(0.0,.35)I(0,)
  mu2[1] ~ dnorm(0.0,.35)I(0,)
  mu1[2] ~ dnorm(0.0,.35)I(,0)
  mu2[2] ~ dnorm(0.0,.35)I(,0)
  # Priors for Variance-covariance matrices for S-C pairs
  # - Test 1
  R1[1:2,1:2] <- inverse(RI1[1:2,1:2])
```

```
  RI1[1,1] <- pow(sigma1[1],2)
  RI1[1,2] <- cov1
  RI1[2,1] <- cov1
  RI1[2,2] <- pow(sigma1[2],2)
  cov1 <- corr*sigma1[1]*sigma1[2]
  sigma1[1] ~ dunif(.001,5)
  sigma1[2] ~ dunif(.001,5)
  corr1 ~ dunif(-1,1)
  # - Test 2
  Identical code for R2 as for R1
  # Obtain estimates for summary statistics
  # - sensitivity and specificity
  SENS[1] <- 1/(1+exp(-mu1[1]))
  SPEC[1] <- 1/(1+exp( mu1[2]))
  SENS[2] <- 1/(1+exp(-mu2[1]))
  SPEC[2] <- 1/(1+exp( mu2[2]))
  # - difference in Se/Sp
  diffSe <- SENS[2]-SENS[1]
  diffSp <- SPEC[2]-SPEC[1]
  # - relative Se/Sp
  RdiffSe <- log(SENS[2]/SENS[1])
  RdiffSp <- log(SPEC[2]/SPEC[1])
  # - relative difference (log-OR) in Se/Sp
  LdiffSe <- log((SENS[2]*(1-SENS[1]))/(SENS[1]*(1-SENS[2])))
  LdiffSp <- log((SPEC[2]*(1-SPEC[1]))/(SPEC[1]*(1-SPEC[2])))
  }
```

Note: Alternatively, for the variance-covariance matrices R1 and R2, we can use a Wishart prior with 3 degrees of freedom: Wishart $\left[\begin{pmatrix} 0.001 & 0 \\ 0 & 0.001 \end{pmatrix}, 3\right]$.

2.2: Example code for Model 2

```
model{
  ### Basic model for results ###
  for (i in 1:nPats){
    # Disease status is provided in the data through the gold standard
    # result GS which is a vector of length nPats with
    # 1=diseased and 0=non-diseased
    status[i] - GS[i]
    # Results for each test follow independent Bernoulli distributions,
    # variable Y[i=patient nr, k=test nr], with probability determined
    # by the study-specific, logit-transformed S and C
    for(k in 1:2){
      Y[i,k] ~ dbern(P[i,k])
      logit(P[i,k]) <- status[i] * alpha[study[i],k] +
```

```
        (1-status[i]) * beta[study[i],k]
    }
  }
### Model for study level results ###
for(j in 1:nStudy){
  # Model for study-specific, logit-transformed S (alpha) and C (beta)
  # mu.site[j,1] is the average (logit) probability of testing
  #   positive in the diseased subjects in site j
  # mu.site[j,2] is the average (logit) probability of testing
  #   positive in the non-diseased subjects in site j
  # rho[j,1] and rho[j,2] are contrasts between S and C in site j
  alpha[j,1] <- mu.site[j,1]+rho[j,1]/2
  alpha[j,2] <- mu.site[j,1]-rho[j,1]/2
  beta[j,1] <- mu.site[j,2]-rho[j,2]/2
  beta[j,2] <- mu.site[j,2]+rho[j,2]/2
  ### Priors for study level results ###
  # Priors for average (logit) probability of testing positive:
  # vague priors that are equivalent to uniform over [0,1] interval
  # Note that 0.35=1/1.69**2
  # Constraints to avoid label switching
  mu.site[j,1] ~ dnorm(0,0.37)I(0,)
  mu.site[j,2] ~ dnorm(0,0.37)I(,0)
  # Priors for contrasts between (logit) S and C
  rho[j,1:2] ~ dmnorm(mu.rho[],R[,])
  }
### Hyper-priors ###
# Means of contrasts between (logit) S and C
mu.rho[1] ~ dnorm(0.0,.001)
mu.rho[2] ~ dnorm(0.0,.001)
# Variance-covariance matrix
R[1:2,1:2] <- inverse(R[1:2,1:2])
RI[1,1] <- pow(sigma[1],2)
RI[1,2] <- cov
RI[2,1] <- cov
RI[2,2] <- pow(sigma[2],2)
cov <- corr*sigma[1]*sigma[2]
sigma[1] ~ dunif(.001,5)
sigma[2] ~ dunif(.001,5)
corr ~ dunif(-1,1)
# Obtain estimates for summary statistics
# - relative difference (log-OR) in Se/Sp
LdiffSe <- mu2[1]
LdiffSp <- mu2[2]
# - average probability of testing positive/negative
mu[1] <- mean(mu.site[,1])
mu[2] <- mean(mu.site[,2])
```

```
  # - sensitivity and specificity
  logit(SENS[1]) <- mu[1]-LdiffSe/2
  SPEC[1] <- 1/(1+exp(mu[2]+LdiffSp/2))
  logit(SENS[2]) <- mu[1]+LdiffSe/2
  SPEC[2] <- 1/(1+exp(mu[2]-LdiffSp/2))
  # - difference in Se/Sp
  diffSe <- SENS[2]-SENS[1]
  diffSp <- SPEC[2]-SPEC[1]
  # - relative Se/Sp
  RdiffSe <- log(SENS[2]/SENS[1])
  RdiffSp <- log(SPEC[2]/SPEC[1])
  }
```

2.3: Example code for Model 3

Model 3 is identical to Model 5 apart from the fact that disease status is provided as data through the gold standard (status[i] − GS[i]).

2.4: Example code for Model 4

Example code for 4 tests.

```
model{
  ### Basic model for results ###
  # The total number of patients is specified as variable nPats #
  for (i in 1:nPats){
    # Disease status follows a Bernouilli distribution, with a
    # study-specific prevalence
    status[i] ~ dbern(prevalence[study[i]])
    # Results for each test follow independent Bernoulli distributions,
    # variable Y[i=patient nr, k=test nr], with probability determined
    # by the study-specific, logit-transformed S and C
    for(k in 1:4){
      Y[i,k] ~ dbern(P[i,k])
      }
    logit(P[i,1]) <- status[i] * alpha1[study[i],1] +
        (1-status[i]) * alpha1[study[i],2]
    logit(P[i,2]) <- status[i] * alpha2[study[i],1] +
        (1-status[i]) * alpha2[study[i],2]
    logit(P[i,3]) <- status[i] * alpha3[study[i],1] +
        (1-status[i]) * alpha3[study[i],2]
    logit(P[i,4]) <- status[i] * alpha4[study[i],1] +
        (1-status[i]) * alpha4[study[i],2]
    }
  ### Model for study level results ###
```

```
  for(j in 1:nStudy){
    # Model for study-specific, logit-transformed S (alpha) and C (beta)
    alpha1[j,1:2] ~ dmnorm(mu1[],R1[,])
    alpha2[j,1:2] ~ dmnorm(mu2[],R2[,])
    alpha3[j,1:2] ~ dmnorm(mu3[],R3[,])
    alpha4[j,1:2] ~ dmnorm(mu4[],R4[,])
    ### Priors for study level results ###
    # Prior for prevalences
    prevalence[j] ~ dbeta(1,1)
    }
  # Priors for average (logit) probability of testing positive:
  # vague priors that are equivalent to uniform over [0,1] interval
  # Note that 0.35=1/1.69**2
  # Constraints to avoid label switching
  mu1[1] ~ dnorm(0.0,.35)I(0,)
  mu2[1] ~ dnorm(0.0,.35)I(0,)
  mu3[1] ~ dnorm(0.0,.35)I(0,)
  mu1[2] ~ dnorm(0.0,.35)I(,0)
  mu2[2] ~ dnorm(0.0,.35)I(,0)
  mu3[2] ~ dnorm(0.0,.35)I(,0)
  # Priors for average (logit) S and 1 - C for reference tests:   #
informative priors
  mu4[1] ~ dnorm(2.5,16)I(0,)
  mu4[2] ~ dnorm(-3.5,40)I(,0)
  # Priors for Variance-covariance matrices for S-C pairs
  # - Test 1
  R1[1:2,1:2] <- inverse(RI1[1:2,1:2])
  RI1[1,1] <- pow(sigma1[1],2)
  RI1[1,2] <- cov1
  RI1[2,1] <- cov1
  RI1[2,2] <- pow(sigma1[2],2)
  cov1 <- corr*sigma1[1]*sigma1[2]
  sigma1[1] ~ dunif(.001,5)
  sigma1[2] ~ dunif(.001,5)
  corr1 ~ dunif(-1,1)
  # - Test 2-4
  Identical code for R2-R4 as for R1
  # Obtain estimates for summary statistics
  # - sensitivity and specificity
  SENS[1] <- 1/(1+exp(-mu1[1]))
  SPEC[1] <- 1/(1+exp( mu1[2]))
  SENS[2] <- 1/(1+exp(-mu2[1]))
  SPEC[2] <- 1/(1+exp( mu2[2]))
  # - difference in Se/Sp
  diffSe <- SENS[2]-SENS[1]
  diffSp <- SPEC[2]-SPEC[1]
```

```
  # - relative Se/Sp
  RdiffSe <- log(SENS[2]/SENS[1])
  RdiffSp <- log(SPEC[2]/SPEC[1])
  # - relative difference (log-OR) in Se/Sp
  LdiffSe <- log((SENS[2]*(1-SENS[1]))/(SENS[1]*(1-SENS[2])))
  LdiffSp <- log((SPEC[2]*(1-SPEC[1]))/(SPEC[1]*(1-SPEC[2])))
  }
```

2.5: Example code for Model 5

Example code for 4 tests.

```
model{
  ### Basic model for results ###
  for (i in 1:nPats){
    # Disease status follows a Bernouilli distribution, with a
    # study-specific prevalence
    status[i] ~ dbern(prevalence[study[i]])
    # Results for each test follow independent Bernoulli distributions,
    # variable Y[i=patient nr, k=test nr], with probability determined
    # by the study-specific, logit-transformed S and C
    for(k in 1:4){
      Y[i,k] ~ dbern(P[i,k])
      logit(P[i,k]) <- status[i] * alpha[study[i],k] +
            (1-status[i]) * beta[study[i],k]
      }
    }
  ### Model for study level results ###
  for(j in 1:nStudy){
    # Model for study-specific, logit-transformed S (alpha) and C (beta)
    # mu.site[j,1] is the average (logit) probability of testing positive
    #   in the diseased in site j
    # mu.site[j,2] is the average (logit) probability of testing positive
    #   in the non-diseased in site j
    # rhoa[j,k] and rhob[j,k] are contrasts between S and C by site
    # Test 4 is the reference
    alpha[j,1] <- mu.site[j,1]+3*rhoa[j,1]/4-rhoa[j,2]/4-rhoa[j,3]/4
    alpha[j,2] <- mu.site[j,1]-rhoa[j,1]/4+3*rhoa[j,2]/4-rhoa[j,3]/4
    alpha[j,3] <- mu.site[j,1]-rhoa[j,1]/4-rhoa[j,2]/4+3*rhoa[j,3]/4
    alpha[j,4] <- mu.site[j,1]-rhoa[j,1]/4-rhoa[j,2]/4-rhoa[j,3]/4
    beta[j,1] <- mu.site[j,2]+3*rhob[j,1]/4-rhob[j,2]/4-rhob[j,3]/4
    beta[j,2] <- mu.site[j,2]-rhob[j,1]/4+3*rhob[j,2]/4-rhob[j,3]/4
    beta[j,3] <- mu.site[j,2]-rhob[j,1]/4-rhob[j,2]/4+3*rhob[j,3]/4
    beta[j,4] <- mu.site[j,2]-rhob[j,1]/4-rhob[j,2]/4-rhob[j,3]/4
    ### Priors for study level results ###
```

```
  # Prior for prevalence
  prevalence[j] ~ dbeta(1,1)
  # Priors for average probability of testing positive in diseased
  #   and non-diseased
  # Note: non-modeled probabilities with independent, low-information
  #   priors
  mu.site[j,1] ~ dnorm(2,1)I(0,)
  mu.site[j,2] ~ dnorm(-2,1)I(,0)
  # Priors for contrasts between (logit) S and C: Independent
  # multivariate priors for contrasts between Ss and Cs
  rhoa[j,1:3] ~ dmnorm(mu.rhoa[],R1[,])
  rhob[j,1:3] ~ dmnorm(mu.rhob[],R2[,])
  }
### Hyper-priors ###
# Means of contrasts between (logit) S and C
for(k in 1:3){
  mu.rhoa[k] ~ dnorm(0.0,.001)
  mu.rhob[k] ~ dnorm(0.0,.001)
  }
# Variance-covariance matrices for contrasts between (logit) S
R1[1:3,1:3] <- inverse(RI1[1:3,1:3])
RI1[1,2] <- cov1[1]
RI1[1,3] <- cov1[2]
RI1[2,1] <- cov1[1]
RI1[2,3] <- cov1[3]
RI1[3,1] <- cov1[2]
RI1[3,2] <- cov1[3]
cov1[1] <- corr1[1]*sigma1[1]*sigma1[2]
cov1[2] <- corr1[2]*sigma1[1]*sigma1[3]
cov1[3] <- corr1[3]*sigma1[2]*sigma1[3]
for(k in 1:3){
  sigma1[k] ~ dunif(.001,5)
  corr1[k] ~ dunif(-1,1)
  RI1[k,k] <- pow(sigma1[k],2)
  }
# Variance-covariance matrices for contrasts between (logit) C
Identical code for R2 as for R1
# Obtain estimates for summary statistics
# - average probability of testing positive/negative
mu[1] <- mean(mu.site[,1])
mu[2] <- mean(mu.site[,2])
# - relative difference (log-OR) in Se/Sp
LdiffSe <- mu.rhoa[2]-mu.rhoa[1]
LdiffSp <- mu.rhob[1]-mu.rhob[2]
# - sensitivity and specificity
logit(SENS[1]) <- mu[1]+3*mu.rhoa[1]/4-mu.rhoa[2]/4-mu.rhoa[3]/4
```

```
logit(SENS[2]) <- mu[1]-mu.rhoa[1]/4+3*mu.rhoa[2]/4-mu.rhoa[3]/4
SPEC[1] <- 1/(1+exp(mu[2]+3*mu.rhob[1]/4-mu.rhob[2]/4-mu.rhob[3]/4))
SPEC[2] <- 1/(1+exp(mu[2]-mu.rhob[1]/4+3*mu.rhob[2]/4-mu.rhob[3]/4))
# - difference in Se/Sp
diffSe <- SENS[2]-SENS[1]
diffSp <- SPEC[2]-SPEC[1]
# - relative Se/Sp
RdiffSe <- log(SENS[2]/SENS[1])
RdiffSp <- log(SPEC[2]/SPEC[1])
}
```

Note: Instead of uniform priors, bounded normal priors can be used for sigma (e.g., sigma1[k] ~ dnorm(0,1)I(.001,5)) and corr (e.g., corr1[k] ~ dnorm(0,25)I(-1,1)). This can improve convergence and avoid computational problems, by placing higher prior probabilities on lower variances between studies and lower correlations among tests.

2.6: Possible Structures for Variance-Covariance Matrices

A general variance-covariance matrix for model 3 is complex. For exploration of this model we can use a simplified variance-covariance structure as an initial approximation and subsequently assess the effects of relaxing these simplifying assumptions. As a working model, a diagonal matrix can be used, corresponding to independence among all δ_{Sij} and δ_{Cij}. Another possibility is to assume homogeneity of variance of the θ_{Sij} and of the θ_{Cij}, the true logit(S_{ji}) and logit(S_{ji}), while ignoring the correlation within $\theta_{Sij} - \theta_{Cij}$. This leads to equality of the variances of the δ_{Sij} and of the δ_{Cij} and in addition to equality of the correlation coefficients between each pair (δ_{Sih},δ_{Sik}), $h \neq k$, $h, k \in 1 \ldots (J-1)$. This results in the following block diagonal matrix as a working model:

$$
\Sigma = \begin{pmatrix}
\sigma^2_{\delta_S} & \rho_{\delta_S}\sigma_{\delta_S} \cdots & \rho_{\delta_S}\sigma_{\delta_S} 0 & 0 \cdots & 0 \\
\rho_{\delta_S}\sigma_{\delta_S} & \sigma^2_{\delta_S} \cdots & \rho_{\delta_S}\sigma_{\delta_S} 0 & 0 \cdots & 0 \\
\vdots & \vdots \ddots & \vdots\vdots & \vdots \ddots & \vdots \\
\rho_{\delta_S}\sigma_{\delta_S} & \sigma^2_{\delta_S} \cdots & \rho_{\delta_S}\sigma_{\delta_S} 0 & 0 \cdots & 0 \\
0 & 0 \cdots & 0 \sigma^2_{\delta_C} & \rho_{\delta_C}\sigma_{\delta_C} \cdots & \rho_{\delta_C}\sigma_{\delta_C} \\
0 & 0 \cdots & 0 \rho_{\delta_C}\sigma_{\delta_C} & \sigma^2_{\delta_C} \cdots & \rho_{\delta_C}\sigma_{\delta_C} \\
\vdots & \vdots \ddots & \vdots\vdots & \vdots \ddots & \vdots \\
0 & 0 \cdots & 0 \rho_{\delta_C}\sigma_{\delta_C} & \sigma^2_{\delta_C} \cdots & \rho_{\delta_C}\sigma_{\delta_C}
\end{pmatrix}
$$

with $\rho_{\delta_S} \equiv \rho_{\delta_C} \equiv \frac{1}{2}$ [34]. Starting from the working model, the assumptions can be relaxed for example by allowing the $\sigma^2_{\delta_{Sj}}$, $\sigma^2_{\delta_{Cj}}$, $\rho_{\delta_{Skj}}$, and $\rho_{\delta_{Ckj}}$ to vary, while retaining the independence of the $\theta_{Sij} - \theta_{Cij}$.
