# Supplementary material for: A general framework for comparative Bayesian meta-analysis of diagnostic studies
Source: BMC Med Res Methodol. 2015 Aug 28;15:70. doi: 10.1186/s12874-015-0061-7 (PMC4552463; doi:10.1186/s12874-015-0061-7)

Additional file 3 — Simulation Study of the Modeling Approach

3.1: Introduction

To assess the performance of the different models and to uncover possible bias of combining data without proper control for study specific effects or adjustment for the use of imperfect reference standards, we performed a simulation study using two scenarios.

In each scenario, the aim is to compare two diagnostic tests T_1 and T_2 with sensitivities $S_1 = 90\%$ and $S_2 = 85\%$ and specificities $C_1 = 85\%$ and $C_2 = 90\%$. Comparison between the tests are made by estimating the relative accuracy through the difference in S and C (S_{D12} and C_{D12}), relative S and C (S_{RR12} and C_{RR12}), or the corresponding ORs (S_{OR12} and C_{OR12}).

The first scenario presents a situation where the same imperfect reference test is used in all studies. The second scenario describes the situation where systematic bias may occur through the use of differing reference tests across studies. The two scenarios are described in detail below.

In each simulation study, we generated 150 sets of 20 diagnostic studies, with each study using two or more of a number of possible diagnostic tests. Each of the simulated diagnostic studies has a moderate sample size of 200 subjects and a disease prevalence of 50%. We analyzed each simulated data set using the models described in the manuscript using the logit for the link function $g(.)$. We present the parameter estimates and standard errors and graphically depict bias in $\hat{S}_{D12}$, $\hat{C}_{D12}$, $\hat{S}_{RR12}$, $\hat{C}_{RR12}$, $\hat{S}_{OR12}$, and $\hat{C}_{OR12}$. We also evaluated the models using coverage probabilities (the proportion of replications in which the credible interval contained the true value) and power (the proportion of replications in which the true difference in S and C between the two tests of interest was detected).

All models were estimated using MCMC methods through Gibbs sampling using OpenBUGS 3.0.3 called from within R 3.0.1 using the BRugs library. For the simulation study convergence was checked using the Gelman-Rubin diagnostic statistic $\hat{R}$, extending simulations until $\hat{R} \leq 1.05$.

3.2: Scenario 1

3.2.1: Setup

In scenario 1, we simulated a setting without systematic bias but where an imperfect reference test is used to assess the diagnostic accuracy of the index tests of interests. Across the 20 diagnostic studies in the simulated meta-analysis there are 3 index tests. T_1 and T_2 are the index tests of interest as described above. T_3 is a third, less accurate index test, with $S_3 = 80\%$ and $C_3 = 80\%$. The reference test, T_4, is a reference standard similar to a parasitological technique or culture in infectious diseases with high specificity (C_4=95%) but lower sensitivity (S_4=80%). We allowed the study-specific S_{ij} and C_{ij} of all tests to vary across studies using (0, .5) normal distributions on the logit scale and induced a correlation ($\rho = 0.5$) between S_{i2} and S_{i4}.

Each simulated diagnostic study uses two or more of 4 possible tests. The availability of the tests is shown in Table 1. In 10 of the 20 studies (studies 1 to 10) a direct comparison between T_1 and T_2 was possible. In five studies (11, 12, 16, 17, and 18) an indirect comparison through the third index test T_3 was possible. The remaining studies only offered information on the diagnostic accuracy of either T_1 and T_2 through comparison with the reference test results.

3.2.2: Analysis

We applied the 5 models described in the methods section and listed in Table 2 of the manuscript to each simulated meta-analysis. Models 1 to 3, that rely on the assumption that a perfect reference test is used, were calculated using the true disease status as reference test and also using the imperfect reference standard T_4. For each analysis, we included only individual basic studies that were informative of the contrast of interest, T_1 versus T_2. This includes only direct comparisons (10 of 20 simulated studies) for model 2, studies which include at least 2 of T_1, T_2 and T_3 for model 3 in simulation study 1 (15 of 20 simulated studies), and all simulated studies in models 1, 4 and 5. In all models, we ignored the correlation between T_2 and the reference test T_4.

Uninformative priors were used for hyperparameters related to the index tests (T_1, T_2, and T_3). Likely in practice some information is available on the diagnostic accuracy of the reference tests T_4, therefore we used informative priors for S_4 and C_4 that were consistent with the simulated values. Specifically, normal priors were provided that indicated with 95% certainty that the average S_4 was in the interval [70%, 95%] and the average C_4 in the interval [90%, 99%].

3.2.3: Results

Table 2 shows the estimates and standard errors of the parameters of interest; Table 3 shows the coverage probabilities and the observed power to detect the difference of 5% in S and C between T_1 and T_2. Figure 1 presents the bias in $\hat{S}_{D12}$ and $\hat{C}_{D12}$ (Figure 1.a), $\hat{S}_{RR12}$ and $\hat{C}_{RR12}$ (Figure 1.b), $\hat{S}_{OR12}$ and $\hat{C}_{OR12}$ (Figure 1.c).

When a true gold standard reference test was available, models 1 to 3 provided unbiased estimates of the S_j and C_j. The contrasts in S_j and C_j expressed as an OR, difference in proportions, or RR were also estimated without bias(Table 2, 'Using Gold Standard' columns). Coverage probabilities were close to 95% (Table 3, 'Using Gold Standard' columns). Model 1 which takes in to account all studies had the smallest standard error and consequently had the highest power to detect a difference in S and C between T_1 and T_2.

In case T_4 was taken as reference standard, the S and especially C of both index tests were underestimated (Table 2, 'Using Imperfect Reference Standard' columns), with very low coverage probabilities (Table 3, 'Using Imperfect Reference Standard' columns). The coverage probabilities for the contrasts between T_1 and T_2 were however better, especially for S_{D12}, C_{D12} (Figure 1.a, models 1-3), S_{RR12}, and C_{RR12} (Figure 1.b, models 1-3). Only substantial bias was observed when estimating C_{OR12} (Figure 1.c, models 1-3), the contrast in specificities between T_1 and T_2 expressed as an odds-ratio, with coverage probabilities below 80% (Table 3, 'Using Imperfect Reference Standard' columns).

Allowing for an imperfect reference resulted in generally unbiased estimates of S and C and of the contrasts between T_1 and T_2 (Table 2 and Figure 1: models 4 and 5). Coverage probabilities were close to 95% for the contrasts between T_1 and T_2 (Table 3: models 4 and 5). The latent class approach appeared to have removed the bias in estimating C_{OR12} that was induced by using the imperfect reference standard T_4 as gold standard.

3.3: Scenario 2

3.3.1: Setup

In scenario 2, we simulated the situation of two index tests which are assessed in primary studies that tend to use different reference standards. In this case T_3 is a highly specific but less sensitive reference standard with $S_3 = 80\%$ and $C_3 = 95\%$ and T_4 is a highly sensitive but less specific reference standard with $S_4 = 95\%$ and $C_4 = 80\%$. We again allowed the S_{ij} and C_{ij} of all tests to vary across studies using (0, .5) normal distributions on the logit scale. We created the possibility of systematic bias by using T_3 as reference test in preference when index test T_1 was assessed and using T_4 as reference test in preference when index test T_2 was assessed. In 5 of the 10 studies which allowed direct comparisons T_3 was the reference standard, in the other 5 studies T_4 was the reference standard. When only T_1 was assessed, and not T_2, T_3 was the reference standard. When only T_2 was assessed, and not T_1, T_4 was the reference standard (Table 4).

3.3.2: Analysis

As the aim of this scenario was to assess the effects of differing imperfect reference standards, we did not analyze this data for the case a true gold standard was available. Models 4.i and 5.i were fitted assuming that the reference tests T_3 and T_4 could differ in diagnostic accuracy. Model 4.ii and 5.ii were fitted under the assumption that T_3 and T_4 were equal. This means that models 4.i and 5.i corresponded to the situation where the researchers knew of the differences in reference standard used across studies and that the variation in reference standard was thus a known source of bias. Models 4.ii and 5.ii corresponded to the situation that researchers were unaware of the differences in reference standards among studies and that consequently the variation in reference standard was an unknown source of bias.

Uninformative priors were used for hyperparameters related to the index tests (T_1 and T_2) and informative priors for hyperparameters related to the reference tests (T_3 and T_4).

3.3.3: Results

Table 5 show the estimates and standard errors of the parameters of interest; Table 6 shows the coverage probabilities and the observed power to detect the difference of 5% in S and C between T_1 and T_2. Figure 2 presents the bias in $\hat{S}_{D12}$ and $\hat{C}_{D12}$ (Figure 2.a), $\hat{S}_{RR12}$ and $\hat{C}_{RR12}$ (Figure 2.b), $\hat{S}_{OR12}$ and $\hat{C}_{OR12}$ (Figure 2.c).

When T_3 and T_4 were assumed to be perfect reference standards and limiting the analysis to direct comparisons between index tests T_1 and T_2 (model 2), we obtained results similar to scenario 1. The differences in accuracy between T_1 and T_2 in terms

of S_{OR12} and C_{OR12} were underestimated (Table 5 and Figure 2.c: model 2), while estimates of S_{D12}, C_{D12}, S_{RR12}, and C_{RR12} were less biased (Table 5 and Figure 2.a-2.b: model 2).

Estimates from model 1, i.e. independently estimating the diagnostic accuracy of T_1 and T_2, resulted in further underestimation of C_1 (Table 5: $\hat{C}_1$=73.6% in model 1, vs 75.4% in model 2), as T_1 tended to be assessed in studies where the less sensitive reference test T_3 was used, resulting in more apparent false positives for T_1. Similarly in this analysis, S_2 was more strongly underestimated (Table 5: $\hat{S}_2$=73.6% in model 1, vs 75.4% in model 2), as T_2 tended to be assessed in studies where the less specific reference test T_4 was used resulting in more apparent false negatives for T_2. As T_1 was the more sensitive test in the simulation and T_2 the more specific, this resulted in an overestimation of the differences in diagnostic accuracy between T_1 and T_2. This is apparent for S_{D12} and C_{D12} (Figure 2.a, model 1) and S_{RR12} and C_{RR12} (Figure 2.b, model 1). The bias is not apparent for S_{OR12} and C_{OR12} (Figure 2.c, model 1), likely due to the fact that the two biases describe counteract eachother.

When correcting for the use of imperfect reference tests using LCA (models 4.i and 5.i), unbiased estimates for the differences in diagnostic accuracy between T_1 and T_2 were obtained (Table 5 and Figure 2). If the data were however analyzed ignoring the differences between the reference tests, the differences in diagnostic accuracy between T_1 and T_2 were again overestimated (Table 5 and Figure 2: models 4.ii and 5.ii).

3.4: Conclusions

This simulation study indicated that the proposed models, and especially models 4 and 5, can result in unbiased estimates for the relative accuracy of two tests while allowing for imperfect reference tests in a meta-analysis and correcting for bias due to confounding induced by differences in reference standard. Ignoring some aspects of the data generating mechanism, for example the correlation between T_2 and the reference test, did not lead to noticeable bias. Ignoring differences among the reference tests, did however lead to important bias. Interestingly, when estimating the difference or relative risk in S and C between two tests, incorrectly assuming that the reference test was perfect did not necessarily invalidate the meta-analysis results, especially not when limiting the analysis to direct comparisons only.

Tables and Figures

Study nr.	T_1	T_2	T_3	T_4
1 to 5	X	X	X	X
6 to 10	X	X		X
11 to 12	X		X	X
13 to 15	X			X
16 to 18		X	X	X
19 to 20		X		X

Table 1 Design of the simulation study - scenario 1. Availability of each index tests (T_1, T_2, T_3) and of the reference test T_4 is indicated by X. 150 simulated datasets of 20 diagnostic studies were generated.

	Using Gold Standard			Using Imperfect Reference Standard			Based on Latent Class Analysis	
Parameter	Model 1 Estimate (SE)	Model 2 Estimate (SE)	Model 3 Estimate (SE)	Model 1 Estimate (SE)	Model 2 Estimate (SE)	Model 3 Estimate (SE)	Model 4 Estimate (SE)	Model 5 Estimate (SE)
S_1	89.7 (1.6)	89.7 (1.6)	89.4 (1.7)	84.7 (1.8)	84.7 (1.8)	84.6 (1.8)	89.8 (1.8)	90.6 (2.0)
S_2	84.2 (2.2)	84.2 (2.2)	84.1 (2.2)	79.5 (2.3)	79.4 (2.1)	79.6 (2.1)	84.6 (2.3)	85.7 (2.3)
C_1	84.8 (2.1)	84.9 (2.1)	84.6 (2.3)	71.1 (2.3)	71.2 (1.9)	71.3 (2.1)	84.6 (2.5)	84.9 (2.7)
C_2	89.3 (1.6)	89.5 (1.7)	89.3 (1.6)	76.3 (1.9)	76.6 (1.7)	76.6 (1.7)	89.4 (1.9)	89.6 (2.0)
S_{D12} (%)	-5.5 (2.8)	-5.6 (3.4)	-5.3 (3.2)	-5.2 (3.0)	-5.3 (3.3)	-5.0 (3.1)	-5.2 (2.9)	-4.9 (3.1)
C_{D12} (%)	4.5 (2.7)	4.6 (3.4)	4.7 (3.2)	5.2 (3.0)	5.4 (3.1)	5.3 (3.0)	4.8 (3.1)	4.7 (3.4)
$log(S_{RR12})$	-0.064 (0.032)	-0.065 (0.040)	-0.062 (0.038)	-0.063 (0.037)	-0.065 (0.041)	-0.061 (0.039)	-0.060 (0.033)	-0.056 (0.036)
$log(C_{RR12})$	0.052 (0.032)	0.053 (0.040)	0.054 (0.038)	0.070 (0.041)	0.073 (0.042)	0.072 (0.041)	0.056 (0.036)	0.053 (0.040)
$log(S_{OR12})$	-0.50 (0.25)	-0.51 (0.31)	-0.48 (0.28)	-0.36 (0.20)	-0.36 (0.23)	-0.35 (0.22)	-0.48 (0.27)	-0.50 (0.31)
$log(C_{OR12})$	0.41 (0.24)	0.42 (0.31)	0.42 (0.28)	0.27 (0.16)	0.28 (0.16)	0.28 (0.16)	0.44 (0.28)	0.43 (0.31)

Note: The simulated values are S_1=90%, S_2=85%, S_3=80%, S_4=80%, C_1=85%, C_2=90%, C_3=80%, C_4=95%, -5.0% for S_{D12}, 5.0 for C_{D12}, -0.057 for $log(S_{RR12})$, 0.057 for $log(C_{RR12})$, -0.46 for $log(S_{OR12})$, and 0.46 for $log(C_{OR12})$.

Table 2 Parameter estimates and standard errors from the proposed meta-analytical models applied in the simulation study - scenario 1. Models 1 to 3 are applied both using the true disease status ("Gold Standard" columns) and disease status estimated from the results of T_4 ("Imperfect Reference Standard" columns). Models 4 and 5 allow for the use of imperfect reference standard through latent class analysis.

	Using Gold Standard			Using Imperfect Reference Standard			Based on Latent Class Analysis	
Parameter	Model 1 Coverage	Model 2 Coverage	Model 3 Coverage	Model 1 Coverage	Model 2 Coverage	Model 3 Coverage	Model 4 Coverage	Model 5 Coverage
S_1	94.8	90.8	92.4	12.0	12.4	8.1	95.4	96.0
S_2	95.2	89.6	92.4	28.8	29.2	24.7	93.8	95.2
C_1	96.0	92.4	93.6	0.0	0.0	0.0	95.4	97.2
C_2	94.0	89.6	91.2	0.0	0.0	0.0	94.6	97.6
S_{D12}	94.8	94.4	96.0	97.6	94.4	97.2	97.1	96.4
C_{D12}	96.8	95.2	97.6	99.2	95.2	97.6	97.5	97.6
S_{RR12}	94.4	94.0	96.0	97.6	94.0	96.4	96.7	96.4
C_{RR12}	96.0	94.8	98.0	98.4	94.0	96.0	97.1	98.0
S_{OR12}	94.8	96.4	95.2	93.2	94.4	93.9	95.9	96.0
C_{OR12}	96.8	94.8	97.2	80.0	77.2	78.5	97.5	97.2
Power(δ_S)	49.2	39.2	40.0	39.2	35.2	34.8	44.4	33.7
Power(δ_C)	42.0	25.2	32.0	40.0	41.2	40.5	34.4	22.9

Table 3 Power and coverages of the 95% credible intervals for the S and C estimates from the proposed meta-analytical models applied in the simulation study - scenario 1. Models 1 to 3 are applied both using the true disease status ("Gold Standard" columns) and disease status estimated from the results of T_4 ("Imperfect Reference Standard" columns).

Study nr.	T_1	T_2	T_3	T_4
1 to 5	X	X	X	
6 to 10	X	X		X
11 to 15	X		X	
16 to 20		X		X

Table 4 Design of the simulation study - scenario 2. Availability of each index test (T_1, T_2) and of the reference tests (T_3, T_4) is indicated by X. 150 simulated datasets of 20 diagnostic studies were generated.

Parameter	Imperfect Reference Standard Model 1 Estimate (SE)	Model 2 Estimate (SE)	Based on Latent Class Analysis Model 4.i Estimate (SE)	Model 4.ii Estimate (SE)	Model 5.i Estimate (SE)	Model 5.ii Estimate (SE)
S_1	82.0 (2.2)	81.1 (1.9)	89.7 (1.9)	90.3 (1.9)	90.6 (2.0)	91.6 (2.2)
S_2	73.6 (2.6)	75.4 (2.1)	84.4 (2.5)	83.2 (2.6)	85.3 (2.9)	83.0 (3.8)
C_1	73.6 (2.7)	75.4 (2.0)	84.4 (2.5)	83.2 (2.6)	85.3 (2.6)	83.0 (3.9)
C_2	81.7 (2.1)	80.8 (1.7)	89.4 (1.8)	90.3 (1.8)	90.5 (2.1)	91.4 (2.2)
S_{D12} (%)	-8.6 (3.4)	-5.9 (3.3)	-5.6 (3.1)	-7.4 (3.2)	-5.6 (3.4)	-9.0 (4.9)
C_{D12} (%)	8.3 (3.4)	5.2 (3.2)	5.4 (3.0)	6.8 (3.1)	5.0 (3.3)	8.1 (4.9)
$log(S_{RR12})$	-0.112 (0.045)	-0.076 (0.042)	-0.069 (0.036)	-0.086 (0.038)	-0.064 (0.040)	-0.104 (0.058)
$log(C_{RR12})$	0.107 (0.045)	0.067 (0.41)	0.062 (0.035)	0.079 (0.37)	0.058 (0.039)	0.094 (0.058)
$log(S_{OR12})$	-0.51 (0.21)	-0.35 (0.20)	-0.54 (0.29)	-0.67 (0.29)	-0.54 (0.33)	-0.85 (0.45)
$log(C_{OR12})$	0.49 (0.20)	0.31 (0.19)	0.48 (0.27)	0.62 (0.29)	0.49 (0.32)	0.76 (0.44)

Note: The simulated values are S_1=90%, S_2=85%, S_3=80%, S_4=80%, C_1=85%, C_2=90%, C_3=80%, C_4=95%, -5.0% for S_{D12}, 5.0 for C_{D12}, -0.057 for $log(S_{RR12})$, 0.057 for $log(C_{RR12})$, -0.46 for $log(S_{OR12})$, and 0.46 for $log(C_{OR12})$.

Table 5 Parameter estimates and standard errors from the proposed meta-analytical models applied to the simulation study - scenario 2. For models 1 and 2, disease status was estimated from the results of the reference test (T_3 or T_4) ("Imperfect Reference Standard" columns). Models 4 and 5 are applied both assuming it is known that the reference tests differ across studies (model 4.i and model 5.i) and ignoring the difference in reference tests (model 4.ii and model 5.ii). Model 3 is not applied as there is no third index test in the simulation.

Parameter	Imperfect Reference Standard Model 1 Coverage	Model 2 Coverage	Based on Latent Class Analysis Model 4.i Coverage	Model 4.ii Coverage	Model 5.i Coverage	Model 5.ii Coverage
S_1	1.3	0.7	93.7	93.2	96.5	95.8
S_2	0.0	0.0	96.0	91.0	98.8	98.6
C_1	0.7	0.7	96.0	94.7	97.9	99.5
C_2	0.7	0.0	94.4	94.7	97.6	96.5
S_{D12}	88.0	95.2	98.4	91.0	98.8	95.8
C_{D12}	91.3	96.6	95.2	95.5	97.6	97.9
S_{RR12}	83.3	93.9	97.6	90.2	98.8	96.5
C_{RR12}	88.0	96.6	95.2	95.5	98.8	97.2
S_{OR12}	98.7	91.8	97.6	92.5	98.8	95.8
C_{OR12}	98.7	87.8	96.0	95.5	96.3	96.5
Power(δ_S)	76.0	42.2	43.7	62.4	34.1	39.4
Power(δ_C)	74.0	34.7	39.7	60.9	25.6	34.5

Table 6 Power and coverages of the 95% credible intervals for the S and C estimates from the proposed meta-analytical models applied from the proposed meta-analytical models applied in simulation study 2. For models 1 and 2, disease status was estimated from the results of the reference test (T_3 or T_4) ("Imperfect Reference Standard" columns). Models 4 and 5 are applied both assuming it is known that the reference tests differ across studies (model 4.i and model 5.i) and ignoring the difference in reference tests (model 4.ii and model 5.ii). Model 3 is not applied as there is no third index test in the simulation.

Figure 1 Bias in estimates of the contrasts in diagnostic accuracy from the proposed meta-analytical models applied in the simulation study - scenario 1. For models 1 to 3 disease status was estimated from the results of T_4. The plots present the bias in $\hat{S}_{D12}$ and $\hat{C}_{D12}$ (first row), $\hat{S}_{RR12}$ and $\hat{C}_{RR12}$ (second row), $\hat{S}_{OR12}$ and $\hat{C}_{OR12}$ (third row).

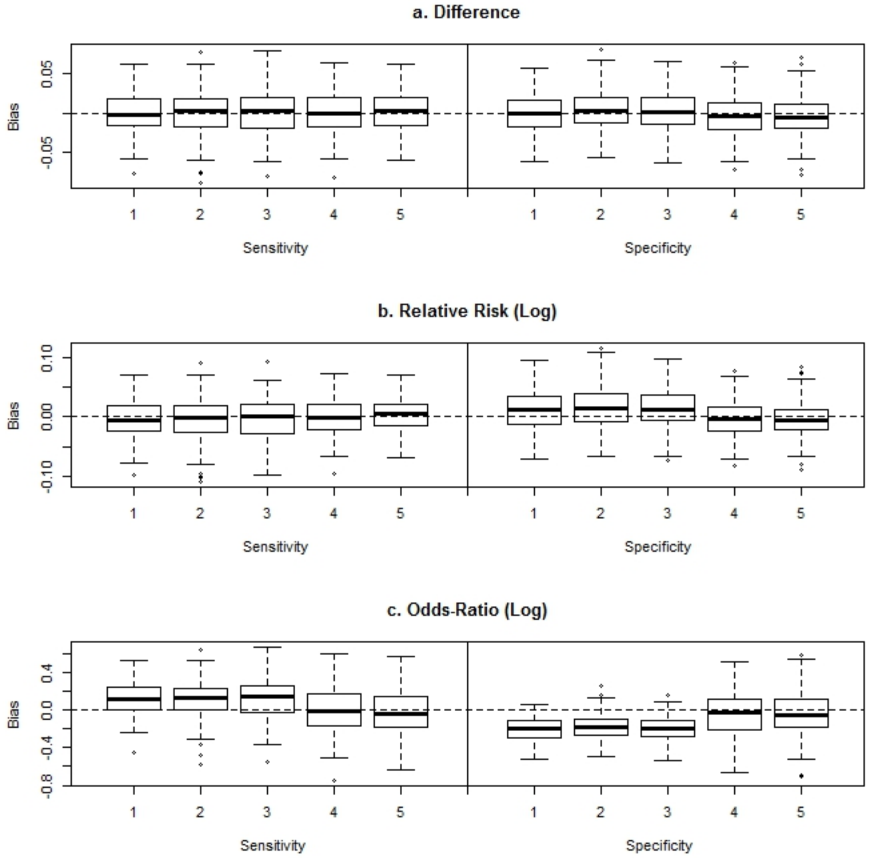

Figure 2 Bias in estimates of the contrasts in diagnostic accuracy from the proposed meta-analytical models applied in the simulation study - scenario 2. Models 4 and 5 were applied both assuming it is known that the reference tests differ across studies (4.i and 5.i) and ignoring the difference in reference tests (4.ii and 5.ii). The plots present the bias in $\hat{S}_{D12}$ and $\hat{C}_{D12}$ (first row), $\hat{S}_{RR12}$ and $\hat{C}_{RR12}$ (second row), $\hat{S}_{OR12}$ and $\hat{C}_{OR12}$ (third row).

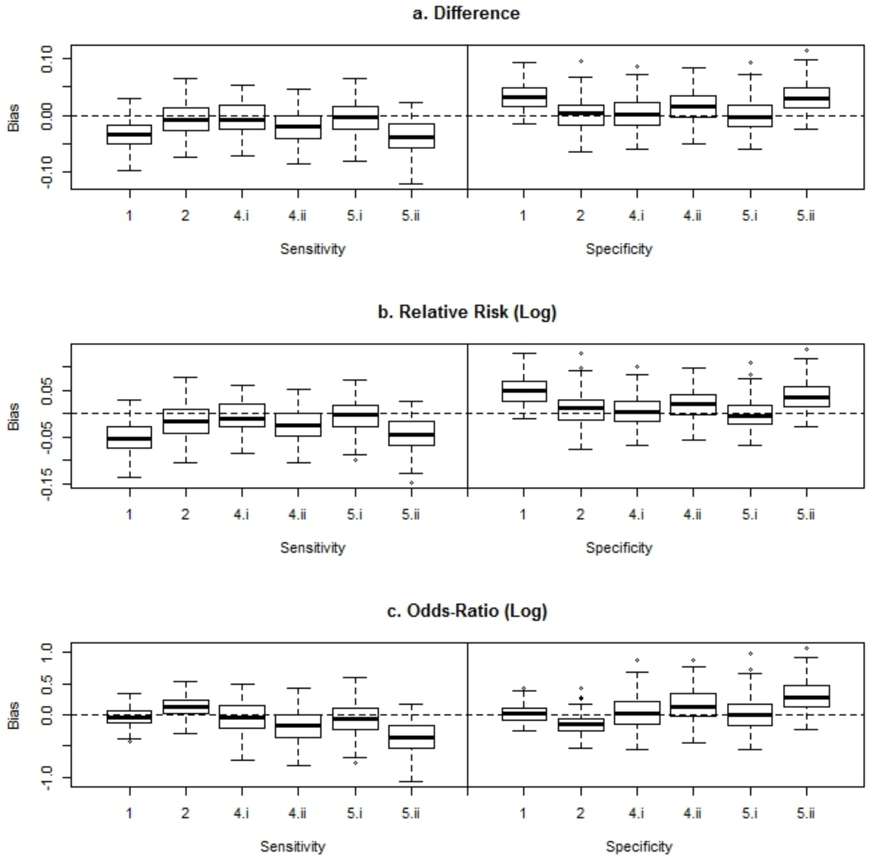

Supplement: Additional file 3 — Simulation Study of the Modeling Approach. (PDF 491 KB) [file 12874_2015_61_MOESM3_ESM.pdf]
