# Supplementary material for: A general framework for comparative Bayesian meta-analysis of diagnostic studies
Source: BMC Med Res Methodol. 2015 Aug 28;15:70. doi: 10.1186/s12874-015-0061-7 (PMC4552463; doi:10.1186/s12874-015-0061-7)
Supplement: Additional file 4 — Visceral Leishmaniasis Data. (PDF 44 KB) [file 12874_2015_61_MOESM4_ESM.pdf]

Additional file 4 — Visceral Leishmaniasis Data

Study Information			Index Tests				Reference Test		Observed
Publication	Country	Region	RK39	DAT	KAtex	IFAT	Spleen	No Spleen	Frequency
Boelaert - 1999	Sudan	2		0		0		0	21
Boelaert - 1999	Sudan	2		0		0		1	1
Boelaert - 1999	Sudan	2		1		0		0	20
Boelaert - 1999	Sudan	2		1		0		1	20
Boelaert - 1999	Sudan	2		1		1		0	6
Boelaert - 1999	Sudan	2		1		1		1	12
Boelaert - 2004	Nepal	1	0	0		0	0		90
Boelaert - 2004	Nepal	1	0	0		0	1		5
Boelaert - 2004	Nepal	1	0	0		1	1		1
Boelaert - 2004	Nepal	1	0	1		0	0		6
Boelaert - 2004	Nepal	1	0	1		0	1		16
Boelaert - 2004	Nepal	1	0	1		1	0		1
Boelaert - 2004	Nepal	1	0	1		1	1		1
Boelaert - 2004	Nepal	1	1	0		0	0		6
Boelaert - 2004	Nepal	1	1	0		0	1		3
Boelaert - 2004	Nepal	1	1	0		1	0		2
Boelaert - 2004	Nepal	1	1	1		0	0		17
Boelaert - 2004	Nepal	1	1	1		0	1		107
Boelaert - 2004	Nepal	1	1	1		1	0		4
Boelaert - 2004	Nepal	1	1	1		1	1		50
Boelaert - 2008	Nepal	1	0	0	0		0		38
Boelaert - 2008	Nepal	1	0	0	0		1		3
Boelaert - 2008	Nepal	1	0	1	0		0		1
Boelaert - 2008	Nepal	1	0	1	1		1		2
Boelaert - 2008	Nepal	1	1	0	0		0		3
Boelaert - 2008	Nepal	1	1	1	0		0		5
Boelaert - 2008	Nepal	1	1	1	0		1		68
Boelaert - 2008	Nepal	1	1	1	1		0		1
Boelaert - 2008	Nepal	1	1	1	1		1		37
Boelaert - 2008	India	1	0	0	0		0		52
Boelaert - 2008	India	1	0	0	1		0		8
Boelaert - 2008	India	1	0	1	0		0		5
Boelaert - 2008	India	1	1	0	0		0		6
Boelaert - 2008	India	1	1	0	0		1		2
Boelaert - 2008	India	1	1	0	1		1		1
Boelaert - 2008	India	1	1	1	0		0		32
Boelaert - 2008	India	1	1	1	0		1		61
Boelaert - 2008	India	1	1	1	1		0		19
Boelaert - 2008	India	1	1	1	1		1		166
Boelaert - 2008	Kenya	2	0	0	0		0		81
Boelaert - 2008	Kenya	2	0	0	0		1		1
Boelaert - 2008	Kenya	2	0	0	1		0		8
Boelaert - 2008	Kenya	2	0	1	0		0		15
Boelaert - 2008	Kenya	2	0	1	0		1		3
Boelaert - 2008	Kenya	2	0	1	1		0		7
Boelaert - 2008	Kenya	2	0	1	1		1		21
Boelaert - 2008	Kenya	2	1	0	0		0		8
Boelaert - 2008	Kenya	2	1	0	1		0		1
Boelaert - 2008	Kenya	2	1	0	1		1		1
Boelaert - 2008	Kenya	2	1	1	0		0		11
Boelaert - 2008	Kenya	2	1	1	0		1		15
Boelaert - 2008	Kenya	2	1	1	1		0		5
Boelaert - 2008	Kenya	2	1	1	1		1		130

Table 1 Real Data Example. Data from a comparative meta-analysis of the RK39 dipstick and direct agglutination test (DAT) for the diagnosis of visceral leishmaniasis. The observed frequency is given for all observed combinations of test results in all primary studies. Other tests: IFAT=indirect fluorescent antibody test, KAtex=latex agglutination test, spleen=parasitological examination of tissue aspirates including spleen sample, no spleen: parasitological examination of tissue aspirates not including spleen sample. 0=negative test result, 1=positive test result, blank=test not performed. Regions: 1=rest of the world, 2=East Africa.

Study Information			Index Tests				Reference Test		Observed
Publication	Country	Region	RK39	DAT	KAtex	IFAT	Spleen	No Spleen	Frequency
Boelaert - 2008	Ethiopia	2	0	0	0		0		7
Boelaert - 2008	Ethiopia	2	0	0	0		1		1
Boelaert - 2008	Ethiopia	2	0	0	1		0		3
Boelaert - 2008	Ethiopia	2	0	1	1		1		4
Boelaert - 2008	Ethiopia	2	1	0	0		0		2
Boelaert - 2008	Ethiopia	2	1	0	1		0		2
Boelaert - 2008	Ethiopia	2	1	1	0		1		5
Boelaert - 2008	Ethiopia	2	1	1	1		1		11
Boelaert - 2008	Sudan	2	0	0	0		0		166
Boelaert - 2008	Sudan	2	0	0	0		1		1
Boelaert - 2008	Sudan	2	0	0	1		0		1
Boelaert - 2008	Sudan	2	0	0	1		1		1
Boelaert - 2008	Sudan	2	0	1	0		0		5
Boelaert - 2008	Sudan	2	0	1	0		1		2
Boelaert - 2008	Sudan	2	0	1	1		0		1
Boelaert - 2008	Sudan	2	0	1	1		1		15
Boelaert - 2008	Sudan	2	1	0	0		0		15
Boelaert - 2008	Sudan	2	1	0	0		1		1
Boelaert - 2008	Sudan	2	1	0	1		0		4
Boelaert - 2008	Sudan	2	1	0	1		1		7
Boelaert - 2008	Sudan	2	1	1	0		0		16
Boelaert - 2008	Sudan	2	1	1	0		1		4
Boelaert - 2008	Sudan	2	1	1	1		0		1
Boelaert - 2008	Sudan	2	1	1	1		1		51
de Assis - 2012	Brazil	1	0	0		0		0	102
de Assis - 2012	Brazil	1	0	0		0		1	1
de Assis - 2012	Brazil	1	0	0		1		0	21
de Assis - 2012	Brazil	1	0	0		1		1	1
de Assis - 2012	Brazil	1	0	1		0		0	4
de Assis - 2012	Brazil	1	0	1		0		1	1
de Assis - 2012	Brazil	1	0	1		1		0	5
de Assis - 2012	Brazil	1	0	1		1		1	11
de Assis - 2012	Brazil	1	1	0		0		1	4
de Assis - 2012	Brazil	1	1	0		1		0	26
de Assis - 2012	Brazil	1	1	1		0		0	7
de Assis - 2012	Brazil	1	1	1		0		1	20
de Assis - 2012	Brazil	1	1	1		1		0	41
de Assis - 2012	Brazil	1	1	1		1		1	160
Toz - 2004	Turkey	1	0			0		0	42
Toz - 2004	Turkey	1	1			0		1	2
Toz - 2004	Turkey	1	1			1		0	1
Toz - 2004	Turkey	1	1			1		1	13
Veeken - 2003	Sudan	2	0	0			0		16
Veeken - 2003	Sudan	2	0	0			1		1
Veeken - 2003	Sudan	2	0	1			1		3
Veeken - 2003	Sudan	2	1	0			0		7
Veeken - 2003	Sudan	2	1	0			1		7
Veeken - 2003	Sudan	2	1	1			0		4
Veeken - 2003	Sudan	2	1	1			1		39
